# Intraspecific variation in seed dispersal of a Neotropical tree and its relationship to fruit and tree traits

**DOI:** 10.1002/ece3.1905

**Published:** 2016-01-25

**Authors:** Carol K. Augspurger, Susan E. Franson, Katherine C. Cushman, Helene C. Muller‐Landau

**Affiliations:** ^1^ Department of Plant BiologyUniversity of IllinoisUrbanaIllinois61801; ^2^ EPA26 W. Martin Luther King Jr. DriveCincinnatiOhio45268; ^3^ Smithsonian Tropical Research InstituteApartado Postal 0843‐03092PanamáRepública de Panamá; ^4^ Department of Ecology and Evolutionary BiologyBrown University80 Waterman St.ProvidenceRhode Island02912

**Keywords:** Crown area, crown height, functional traits, recruitment, seed shadow, tails of distribution, wind dispersal, wing loading

## Abstract

The distribution of wind‐dispersed seeds around a parent tree depends on diaspore and tree traits, as well as wind conditions and surrounding vegetation. This study of a neotropical canopy tree, *Platypodium elegans,* explored the extent to which parental variation in diaspore and tree traits explained (1) rate of diaspore descent in still air, (2) distributions of diaspores dispersed from a 40‐m tower in the forest, and (3) natural diaspore distributions around the parent tree. The geometric mean rate of descent in still air among 20 parents was highly correlated with geometric mean wing loading^1/2^ (*r* = 0.84). However, diaspore traits and rate of descent predicted less variation in dispersal distance from the tower, although descent rate^−1^ consistently correlated with dispersal distance. Measured seed shadows, particularly their distribution edges, differed significantly among six parents (DBH range 62–181 cm) and were best fit by six separate anisotropic dispersal kernels and surveyed fecundities. Measured rate of descent and tree traits, combined in a mechanistic seed dispersal model, did not significantly explain variation among parents in natural seed dispersal distances, perhaps due to the limited power to detect effects with only six trees. Seedling and sapling distributions were at a greater mean distance from the parents than seed distributions; saplings were heavily concentrated at far distances. Variation among parents in the distribution tails so critical for recruitment could not be explained by measured diaspore or tree traits with this sample size, and may be determined more by wind patterns and the timing of abscission in relation to wind conditions. Studies of wind dispersal need to devote greater field efforts at recording the “rare” dispersal events that contribute to far dispersal distances, following their consequences, and in understanding the mechanisms that generate them.

## Introduction

Distance and direction of dispersal affect a seed's fate. Parent fitness depends on the distribution of seed trajectories, that is, on the full seed shadow. Effectively, parents are bet‐hedging; dispersing their offspring to a variety of microenvironments maximizes the chance that some will survive. Not all environments within a seed shadow are equally important. Distance‐ and density‐dependent mortality makes the tails particularly important.

Intraspecific variation in seed shadows and associated variation in parent fitness of a wind‐dispersed species depend on diaspore traits such as wing loading, tree traits such as tree height and crown area, as well as wind conditions and surrounding vegetation that modify local winds. However, few studies have investigated intraspecific variation in seed shadows resulting from wind dispersal, the factors explaining that variation (Nathan et al. 2001; Norghauer et al. 2011), or its consequences for recruitment (Schupp and Fuentes 1995; Nathan and Muller‐Landau 2000). Ballistic‐dispersed (Norghauer and Newbery 2015) and animal‐dispersed species (Clark et al. 2005; Russo et al. 2006; Swamy et al. 2011) have been studied in this context. Interspecific studies have found that a species' dispersal distance can be predicted from simple plant traits, including growth form (a proxy for plant height) and terminal velocity of wind‐dispersed species (Thomson et al. 2011; Tamme et al. 2014).

Field studies supporting the hypothesis that diaspore traits of wind‐dispersed species affect dispersal distance are complicated because of the lack of control of additional factors, including tree height and wind conditions that affect dispersal. Species with variable numbers of seeds per diaspore (and thus variable wing loading and rate of descent) on the same tree can be used to test the hypothesis because all diaspore types are presumably at comparable tree heights and are dispersed under common wind conditions. In two such studies, as seed number per diaspore increased, wing loading^1/2^ and rate of descent increased, while dispersal distance decreased (Augspurger and Hogan 1983; Augspurger 1986a). Another approach is to release diaspores from a tower in the forest, thus controlling for tree release height and wind conditions. In a field study using a series of artificial diaspores with exaggerated variation in diaspore traits and released from a tower, variation in diaspore traits explained variation in dispersal distances and distributions; mass ranged from 1.0 to 1.9 g, area from 9 to 76 cm^2^, and wing loading from 15.3 to 30.6 mPa (Augspurger and Franson 1987, 1993). Likewise, Morse and Schmitt (1985) showed experimentally that intraspecific variation in diaspore morphology of a wind‐dispersed herb produced substantial variation in dispersal capacity. However, Nathan and Muller‐Landau (2000) concluded that intraspecific variation in plant traits is relatively low and that variation in wind conditions, affecting both diaspores within a tree crown and individuals in a population, becomes paramount in explaining most intraspecific variation in dispersal distances.

**Figure 1:**
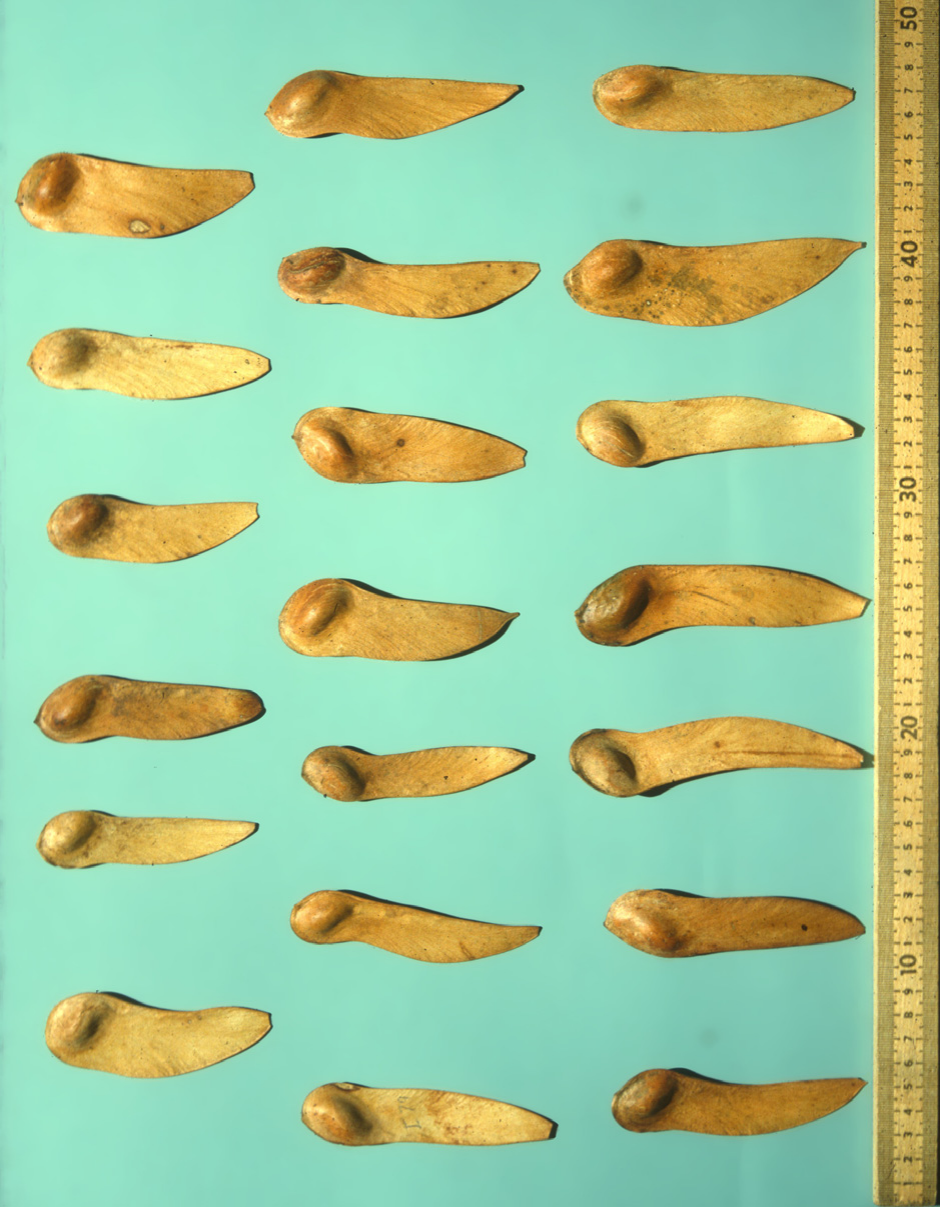
A representative fruit of each of the twenty study trees of *Platypodium elegans* on BCI, Panama. Scale is in centimeters. Photograph: C. Augspurger.

This intraspecific study of *Platypodium elegans,* a wind‐dispersed tropical canopy tree, investigated whether its diaspore (hereafter fruit) and tree traits explain seed shadows and consequences for recruitment. The study capitalized on noticeable variation among parents in fruit traits that affect dispersal (Fig. 1). We compared progressively the fit of predictions based on fruit traits to (1) rate of descent in still air, (2) dispersal distance from a tower under controlled height, wind, and vegetation conditions, and (3) aspects of distributions within seed shadows around individual parent trees (Table 1). Regression models that incorporated both fruit wing loading and tree traits (crown height, crown area) were developed to determine the extent to which these factors predicted various descriptors of seed shadows.

**Table 1:** Overview of the study. Predictor variables were collected sequentially to test their explanatory power in explaining (1) rate of descent in still air, (2) dispersal distributions from the tower, (3) seed shadow descriptors under natural field conditions, and (4) seedling/sapling distributions. N refers to number of parents sampled

Predictors	Responses
Fruit traits (mass, area, wing loading) (Variation among parents (*n* = 20) and Variation between years (*n* = 18))	Rate of descent in still air (Experiment 1) (*n* = 20)
Variation among fruit traits + rate of descent	Dispersal distance from tower (Experiment 2)
Variation among trees in fruit traits + rate of descent (*n* = 20)	Mean (percentiles) dispersal distance from tower (Experiment 2) (*n* = 20)
Wing loading of individual fruits	Dispersal distance from parent tree
Seed dispersal kernels (*n* = 6)	Seed shadow descriptors (*n* = 6)
Dispersal distances from tower (Experiment 3) (*n* = 6)	Seed shadow descriptors (*n* = 6)
Mechanistic model: Rate of descent (Experiment 1) and Tree traits (crown area, height) (*n* = 6)	Seed shadow descriptors (*n* = 6)
Seed distributions (*n* = 6)	Seedling and sapling distributions (*n* = 4–6)

To determine which areas of the seed shadow were most important for recruitment, the study also compared seed versus seedling and sapling distributions. Seedlings of *Platypodium elegans* experienced mortality via distance‐ and density‐dependent fungal pathogens (Augspurger 1983a; Augspurger and Kelly 1984), resulting in low recruitment under and near the parent tree; mortality due to pathogens was nearly total within 20 m of the parent, and less at greater distance, thus supporting the Janzen‐Connell hypothesis (Janzen 1970; Connell 1971). In addition, offspring have enhanced survival and growth in light gaps, where mortality from fungal pathogens is lower than in shade (Augspurger and Kelly 1984); the probability of a parent having some offspring in a light gap increases with greater area of a seed shadow. Therefore, in the analyses, we placed emphasis on identifying factors that explain the tails of the seed shadow as well as its total area.

Our overall prediction was that increasingly complex combinations of predictor variables would be required to explain response variables as the study moved from still air to tower conditions to natural field conditions (Table 1). Our specific predictions were as follows: 1) fruits with lower wing loading^1/2^ have a slower rate of descent in still air and, to a lesser extent, a greater dispersal distance from the field tower; 2) both tree and fruit traits would need to be in regression models to explain dispersal distances within natural seed shadows, as larger trees would generate greater dispersal distances; 3) unmeasured factors, including abscission strength and wind conditions, would become increasingly important, especially in explaining edges and area of the seed shadow, known to be important for recruitment in this species.

## Materials and Methods

### Study site, study species, and focal parent trees

The study site, Barro Colorado Island, Panama, has a semideciduous forest with a dry season (December–April) with trade winds blowing consistently from N to NE. The study species, *Platypodium elegans* J. Vogel (Leguminosae: Papilionoideae), is a canopy tree that is deciduous in the dry season when its wind‐borne fruits are dispersed primarily from late January to mid‐March. The dispersal unit is a (usually) 1‐seeded fruit, a dry, indehiscent samara born on a 2‐cm stipe (see Methods S1). The rare 2‐seeded fruits were not included in this study (Augspurger 1986a). During dispersal, its wing produces lift as it spins tightly around the seed at the distal end of the fruit (nonrolling autogyro) (Augspurger 1986b). Reproductive trees range from 20 cm (Muller‐Landau et al. 2008) to 139 cm at 2 m height (BCI census unpublished data). Twenty reproductive trees were selected for study, all well isolated from conspecifics. Eighteen were focal trees in both 1980 and 1983 (henceforth Year 1 and Year 2); two additional parents were collected in only Year 2.

### Measure fruit traits and rate of descent

For each parent in each year, 100 fruits were collected haphazardly in areas of high density between ≈ 8 m, just beyond the crown edge, and ≈ 15 m, near the peak distance of fruits south (i.e., downwind) of the parent trunk. Fruit mass was determined shortly after collection on a top‐loading balance. Fruit area was determined in Year 1 by tracing fruit shape on gridded paper and in Year 2 using a leaf area meter (average of three readings). Wing loading was calculated as mass/area. Rate of descent in still air was measured for each fruit of Year 2 by determining the time required to fall through 27 m of still air in a theater (Experiment 1) (see Methods S2). To translate these values into implications for dispersal distances, we calculated expected standard dispersal distance from a 40‐m tower under mean wind speeds of 1 m/s as 40*1/(rate of descent) (Cremer 1977). We analyzed variation in fruit area, mass, wing loading^1/2^, and rate of descent among trees and between years using ANOVAs.

### Experiments to quantify dispersal distance from tower

In two experiments during April of Year 2, fruits were released from a 40‐m meteorological tower in the forest on BCI. Experiment 2 used ≈ 100 fruits measured previously for each of the 20 parents collected in Year 2. Each fruit was individually numbered with a felt‐tip marker pen. Experiment 3 used ≈ 300 (Parents 2, 3, 4, and 5) or 100 (Parents 6 and 7) individually numbered fruits of each of the six parents collected when mapping their seed shadows. All fruits were released simultaneously from trapdoors at the bottoms of five boxes (46 x 31 x 31 cm deep) extended on poles 2 m from the downwind (SW) side of the tower. The wind speed above the canopy at the time of release was 25 km/h (7 m· sec^−1^). From the tower, we identified the maximum area to search for the dispersed fruits in our 100 m x 100 m grid system downwind (south) of the tower. On the day subsequent to the late afternoon drops from the tower, we systematically walked the grid system twice to collect and map fruits by 2.5 x 2.5 m quadrat (see Methods S2).

### Models: Fruit traits predict rate of descent and dispersal distance from tower

We fit several alternative models to explain variation among fruits in the rate of descent in still air (Experiment 1) and dispersal distances from the tower (Experiment 2). To explain fruit‐level variation in rate of descent in still air, we compared six models that included mass, area, and wing loading (see Methods S3 for detailed model descriptions). To explain fruit‐level variation in dispersal distance from the tower, we compared models that included these fruit traits and/or rate of descent (see Methods S3 for detailed model descriptions). We compared the models using Akaike's information criterion (AIC).

We also tested whether fruit‐level relationships explain among‐tree variation in the geometric means, percentiles, and maximum rates of descent (Experiment 1) and dispersal distances (Experiment 2) (see Methods S3). We used Pearson correlation of predicted values with measured values to assess explanatory power.

### Wing loading versus dispersal distance under natural field conditions

For Parent 5 only, 1‐5 fruits were collected at each 1‐m distance on a 50‐m transect S of the parent. Wing loading of each fruit was determined. A simple regression analysis on log‐transformed data evaluated how well an individual fruit's wing loading explained its actual dispersal distance from a single tree.

### Measure seed shadows

For six of the 20 parent trees, seed distributions around the tree (“seed shadows”) were censused in Year 1 in the dry season after dispersal was complete. Fruits were counted in 1‐m^2^ quadrats at each meter along six transects (N, E, SE, S, SW, and W) originating at the tree trunk's edge (see Methods S4). Maximum dispersal distance for each transect was declared if no more fruits were found within the next 20 m. These numbers then formed the basis for seed shadow maps and for estimates of seed shadow areas, contours, and total crop size (see Methods S4).

For each parent tree, we computed mean distance of fruits from the tree trunk, distance of peak density, distances to which various cumulative percentages of fruits were dispersed from the tree trunk (dispersal distance percentiles), and total area of the fruit distribution. Total area of the fruit distribution was estimated by the interpolated curved line outlining the perimeter based on maximum distance of the eight transects.

### Fit of seed dispersal kernel models

We fitted seed dispersal kernel models to describe the observed fruit densities around parent trees. The alternative fitted kernels were the full suite described in Van Putten et al. (2012) (see Methods S4). We compared AIC values to determine the best‐fit kernel. From the best‐fit seed shadow of each parent, we calculated mean dispersal distance of fruits from their point sources within the crown (how far each fruit actually traveled) and mean distance from the parent trunk. Pearson correlations were used to evaluate the consistency among trees between metrics of dispersal distance calculated from measured seed shadows and estimated from modeled dispersal kernels.

### Compare tower versus seed shadow distributions

We assessed the consistency in rankings among parents for dispersal distance percentiles of 25%, 50%, 75%, 90% and 95% within the tower distributions (Experiment 3), within the seed shadow distributions, and between tower and seed shadow distributions using Kendall's coefficient of concordance (see Methods S4).

### Models of fruit and tree traits and seed shadow descriptors

Due to the small number of parent trees (*n* = 6) with measured seed shadows and large number of potential explanatory traits, we used a mechanistic model (WINDISPER‐E; Nathan et al. 2002b) to predict differences in dispersal due to combined fruit and tree traits rather than evaluating each trait separately. The crown height, crown area, and fruit rate of descent measured for each tree were used as inputs to the model (see Methods S5); wind data were sampled from high‐frequency (10 Hz) measurements at the same site during the *Platypodium elegans* dispersal season, and assumed to be the same for each tree (see Methods S5). Pearson correlations were used to evaluate how well fruit and tree trait variation (via the WINDISPER‐E mechanistic model) predicted variation in dispersal around parent trees (see Methods S5). These analyses were considered preliminary as the sample size of six parent trees was low for attempting these intraspecific comparisons.

### Seed/Seedling/Sapling distances from parent trees

For all six parents with measured seed shadows, saplings (older than 1 year and <2 m tall) were mapped within the seed shadow (see Methods S6). For four of these parents, 9‐month seedlings were also mapped (see Methods S6). The percentage of all seeds, seedlings, and saplings at each 5‐m interval from the parent was calculated. In addition, calculations were made of seedlings per seed and saplings per seed as a function of distance and of seedlings per seed and saplings per seed as a function of the dispersal distance percentile.

## Results

### Fruit traits, rate of descent, and dispersal distance from tower

**Figure 2:**
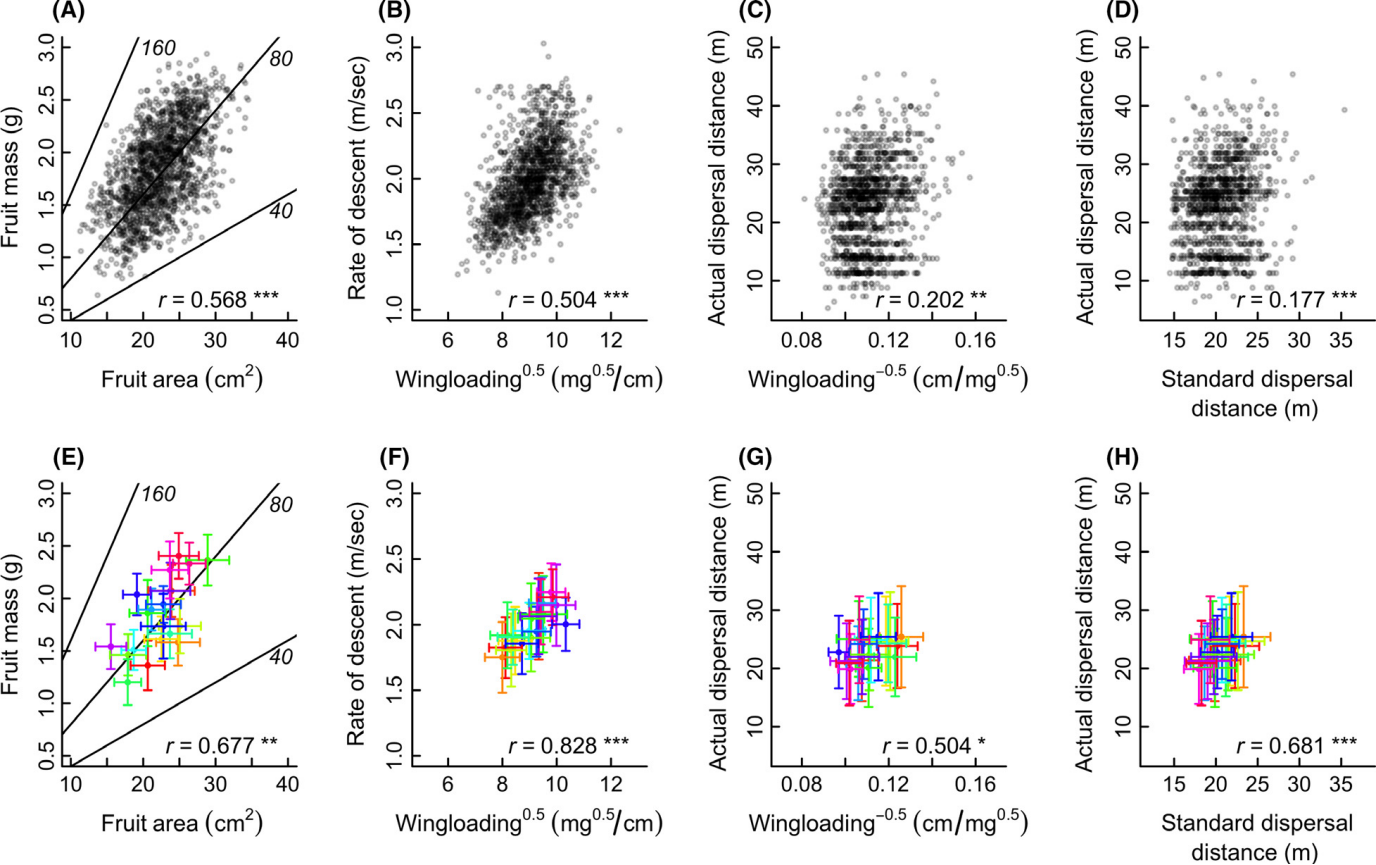
Within‐ and among‐tree variation in fruit traits, rate of descent, and dispersal distance from the tower, and their interrelationships for *Platypodium elegans* on BCI, Panama. At top, variation among fruits, with one point per fruit, with correlation coefficients for the displayed relationships (***P < 0.001, **P < 0.01, *P < 0.05) (*n* ≈ 100 fruits per parent). At bottom, variation among 18 trees, with one star per tree and bars extending to one standard deviation from the mean. Lines showing equal wing loading are included for relationships between fruit mass and area (A, E), with italic values indicating the wing loading value in mg/cm^2^.

**Figure 3:**
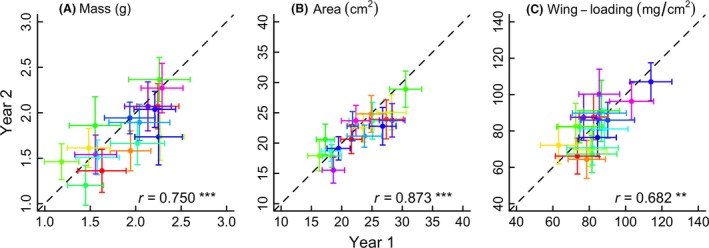
Interannual variation in fruit traits, showing the relationship between trait values in Year 1 (*x*‐axis) and Year 2 (*y*‐axis) for *Platypodium elegans* on BCI, Panama. Relationships for fruit mass (A), fruit area (B), and wing loading (C) are shown. Each point represents the mean trait value of one parent tree with bars representing one standard deviation around the mean (*n* = 18 parent trees). Dashed lines show one‐to‐one relationships. Correlation coefficients for the displayed relationships between means of parent trees are shown (***P < 0.001 and **P < 0.01).

Fruit mass, area, and wing loading^1/2^ varied within and among parents, between years, and with parent–year interactions (Fig. 2A, B, E, F; Table S1). Total variance explained was 20% for mass, 64% for area, and 31% for wing loading^1/2^. Parent effects were much stronger than year effects or parent–year interactions, although extensive overlap in distributions existed among parents (Fig. 2E, F; Table S1). The fruits of the 18 and 20 parents varied in mean mass by 2.0‐fold and 2.2‐fold in Year 1 and Year 2, respectively, 1.9‐fold for mean area in both years, and 1.4‐ and 1.3‐fold for mean wing loading^1/2^ (Table S2). Within parents, fruit traits varied between years, although values were significantly positively correlated between years (Fig. 3, Table S2).

The 20 parents also differed significantly in mean rate of descent in still air of fruits (Experiment 1) and mean distance of fruits from tower (Experiment 2) (each one‐way ANOVA *P *<* *0.0001; Fig. 2F, G; Table S2). The parents differed 1.3‐fold for mean rate of descent and 1.4‐fold for mean distance from tower. Differences among parents in percentiles of dispersal distances were approximately proportional, meaning absolutely larger differences in distribution tails (Fig. S1; Table S3).

### Models: Fruit traits predict rate of descent and dispersal distance from tower

Fruit traits explained substantial variation in rate of descent among fruits (Experiment 1) (Fig. 2B, F, Table 2). The best model to explain among‐fruit variation in rate of descent included both mass and area to fitted powers and explained 27.8% of the variation. The model with wing loading^1/2^ alone to a fitted power explained 27.5% of the variation, while the first principles model of proportionality with wing loading^1/2^ explained 24.2% of the variation; both of these models were significantly poorer fits (ΔAIC of 6.5 and 68.8, respectively). Nonetheless, the first‐principles model of wing loading^1/2^ was an excellent predictor of among‐parent variation in means and percentiles of rate of descent (correlation coefficients of 0.75–0.85 for all percentiles except maximum (0.29)) (Table 3), while the best fruit‐level model of mass and area was also an excellent predictor (correlation coefficients of 0.71–0.88).

**Table 2:** Models explaining fruit‐level variation in rate of descent and dispersal distance of *Platypodium elegans* from the tower on BCI, Panama. *DR* is descent rate (m/sec), *WL* is fruit wing loading (mg/cm^2^), *A* is fruit area (cm^2^), and *M* is fruit mass (g). Adjusted *R* ^2^ values, relative AIC values, and posterior model weights are reported. Models indicated in bold were used to calculate correlations between predicted and observed tree‐level descent rate and dispersal distance (Table 3)

Dependent variable	Model	*R* ^2^	ΔAIC	Weight
Log (descent rate)	**1.478 + 0.383 Log(M) − 0.330 Log(A)**	0.278	0.00	0.963
	−0.953 + 0.371 Log(WL)	0.275	6.55	0.037
	**−1.518 + 0.5 Log(WL)**	0.242	68.81	<0.0005
	0.540 + 0.241 Log(M)	0.157	222.95	<0.0005
	0.839 − 0.052 Log(A)	0.004	464.59	<0.0005
	0.678 *(Null model)*	–	469.02	<0.0005
Log (dispersal distance from tower)	**3.990 − 0.170 Log(WL) − 0.232 Log(DR)**	0.028	0.00	0.420
	2.974 − 0.186 Log(M) + 0.121 Log(A) − 0.225 Log(DR)	0.029	0.50	0.326
	3.344 − 0.119 Log(M) − 0.279 Log(DR)	0.027	1.48	0.200
	4.218 − 0.257 Log(WL)	0.022	6.40	0.017
	2.634 − 0.273 Log(M) + 0.198 Log(A)	0.023	6.40	0.017
	3.329 − 0.358 Log(DR)	0.022	6.82	0.014
	3.413 − 0.027 Log(A) − 0.360 Log(DR)	0.021	8.56	0.006
	3.193 − 0.184 Log(M)	0.016	13.77	<0.0005
	**5.284 − 0.5 Log(WL)**	0.002	29.08	<0.0005
	3.087 *(Null model)*	–	32.16	<0.0005
	3.115 − 0.009 Log(A)	<0.0005	34.14	<0.0005
	**3.763 − Log(DR)**	<0.0005	90.80	<0.0005

**Table 3:** Pearson correlations between modeled and observed tree‐level statistics for descent rate and dispersal distance of *Platypodium elegans* on BCI, Panama. For measured descent rate and measured dispersal distance from tower, predictions are based on measured fruit traits and models fitted to fruit‐level data (Table 2). For mechanistic model dispersal distance, field measurements are compared to predicted distance from the trunk, and fitted kernel measurements are compared to predicted distance from a point source within the crown. Significance of correlations = ***P < 0.001, **P < 0.01, *P < 0.05, and (*)*P* < 0.1

	Mean	Median	75th percentile	90th percentile	95th percentile	Maximum
	Measured descent rate					
Best fruit model	0.846***	0.854***	0.875***	0.813***	0.840***	0.705***
First principles: wing loading^0.5^	0.837***	0.831***	0.85***	0.762***	0.752***	0.286
	Measured dispersal distance from tower					
Best fruit model	0.495*	0.51*	0.618**	0.66**	0.611**	0.593**
First principles: wing loading^0.5^	0.386(*)	0.437(*)	0.571**	0.533*	0.516*	0.470*
First principles: descent rate^−1^	0.608**	0.556*	0.623**	0.726***	0.638**	0.482*
	Mechanistic model dispersal distance					
Field measurements	0.337	0.44	0.161	0.207	0.453	0.739(*)
Fitted kernel	0.025	0.044	0.071	0.075	0.068	0.264

In contrast, neither fruit traits nor rate of descent (Experiment 1) nor their combination could explain much among‐fruit variation in dispersal distance from the tower (Experiment 2) (Fig. 2C, D, G, H; Table 2). The best model for among‐fruit variation included both wing loading and rate of descent to fitted powers and explained only 2.8% of the variation in dispersal distance (Table 2). First principles models of proportionality with wing loading^−1/2^ or the inverse of descent rate explained essentially no variation (0.2% and <0.005%, respectively). These models did substantially better at explaining among‐tree variation in means and percentiles of dispersal distance (correlations of 0.48–0.73 and 0.39–0.57 for the model based on descent rate or wing loading, respectively), though not all correlations were significant at *P* < 0.05 (Table 3). The best fruit‐level model had correlations of 0.50–0.66.

### Wing loading versus dispersal distance under natural field conditions

Dispersal distance of individual fruits of Parent 5 increased significantly with decreasing wing loading^1/2^, but only 4.7% of the variation in distance among fruits was explained by this fruit trait (*Y* = 68.297−0.186X, *R* ^2 ^= 0.047, *n* = 99, *P* < 0.03). When fruits not dispersed (under crown of parent) were excluded from the analysis, the relationship was nonsignificant.

### Seed shadows

**Figure 4:**
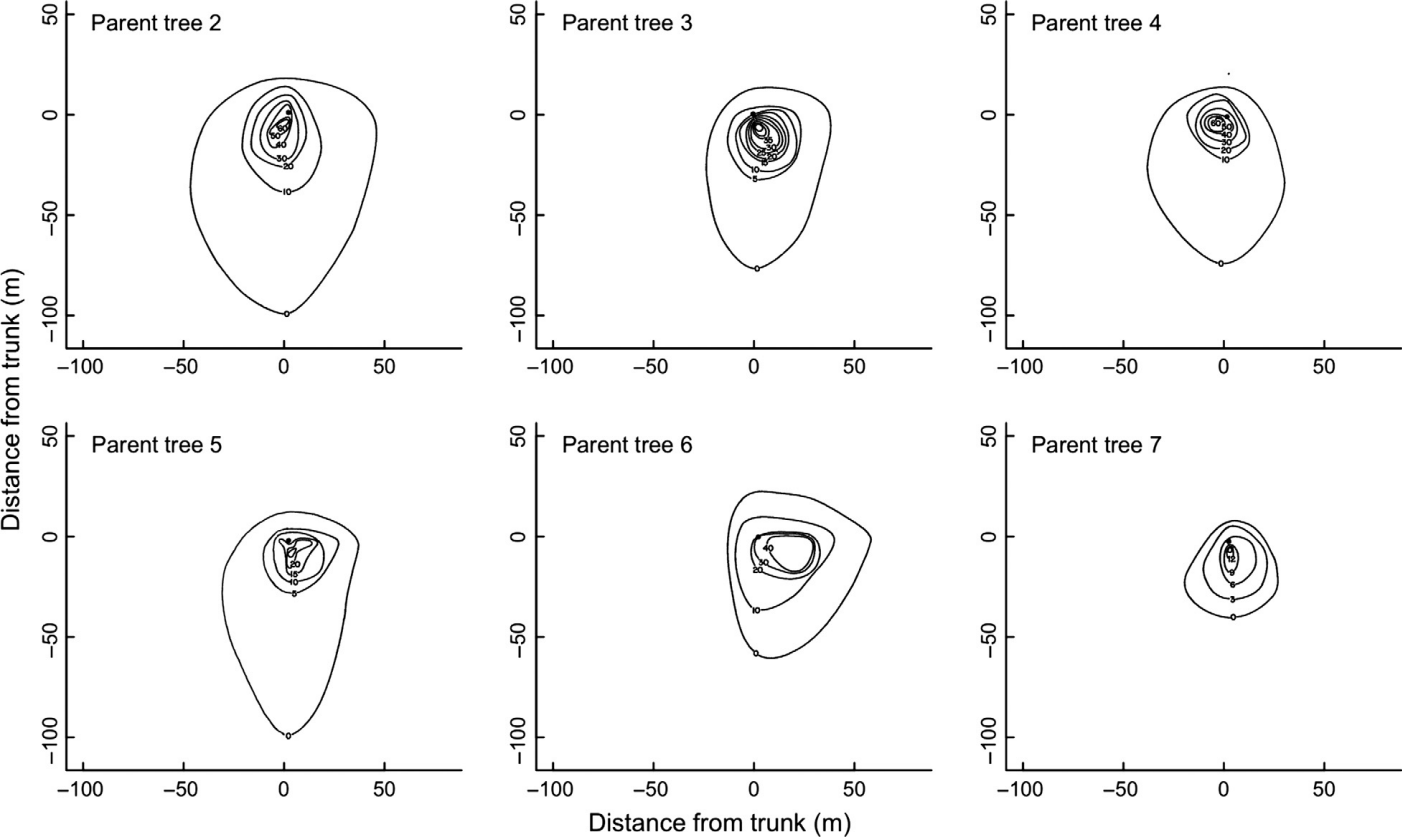
Mapped seed shadows for six parent trees of *Platypodium elegans* on BCI, Panama. A solid black circle indicates the location of the parent tree trunk. Contour intervals represent seed density per m^2^ (the same as in Fig. 5). North direction is toward the top of figures.

Seed shadows differed among the six parents in shape, area, distribution of density throughout the shadow, and dispersal distance distribution (Fig. 4). Parents differed 4.9‐fold and 9.1‐fold in seed shadow area and crop size, respectively (Table 4). All shadows were asymmetric, with most fruits dispersed south of the parent tree. Among parents, 7–23% of seeds were nondispersed, that is, within the tree crown radius (Table 4). Peak density ranged among parents from 12 to 60 fruits per m^2^, and its location ranged from 7 to 24 m from the parent tree (Table 4). Mean estimated dispersal distances of all fruits (not just sampled fruits) differed significantly among parents (one‐way ANOVA F = 1,718.8, df = 5, 165,258, *P* < 0.05; Table 4, Methods S3). In contrast to the tower results, actual mean distance was not significantly correlated with expected mean distance calculated from rate of descent in still air and mean crown height (*r* = 0.105, *n* = 6, n.s.) (Table 4).

**Table 4:** Tree size, fruit rate of descent, and seed shadow statistics for each of six parents of *Platypodium elegans* on BCI, Panama

	Parent 2	Parent 3	Parent 4	Parent 5	Parent 6	Parent 7
DBH (cm)	92	77	181	90	72	62
Crown radius^a^ (m)	8.7	5.0	7.3	8.6	8.3	6.0
Crown area (m^2^)	232	95	167	216	201	98
Tree height (m)	35	30	34	36	30	30
Mean crown height (m)	30.5	28	28.5	31	27	26
Rate of descent (m·sec^−1^) Mean (SD)	2.1 (0.3)	1.8 (0.3)	1.9 (0.3)	1.8 (0.3)	1.9 (0.3)	2.0 (0.3)
Expected standard dispersal distance^b^ (m) Mean (SD)	15.2 (2.4)	16.3 (2.3)	15.4 (1.9)	17.5 (2.5)	14.5 (2.1)	12.9 (1.7)
Dispersal distance from tower (m) Mean (SD)	22.3 (7.5)	23.1 (6.4)	22.3 (6.1)	24.2 (6.5)	23.0 (6.6)	23.5 (7.1)
Dispersal distance from trunk (m) Mean (SD)	25.4 (19.2)	20.4 (11.6)	16.3 (13.2)	17.7 (11.1)	22.0 (9.7)	16.5 (8.2)
Estimated total crop size	48,993	21,719	24,742	14,526	50,680	5566
Total seed shadow area (m^2^)	7666	2912	4256	5531	4137	1572
Proportion of nondispersed (%)	18.0	7.3	27.8	7.7	22.6	11.7
Distance of peak fruit number (m)	14	21	7	9	24	11

a Geometric mean of six radii.

b Calculated under the assumption of a 40 m height and 1 m/sec wind speed during dispersal.

### Fit of seed dispersal kernel models

**Figure 5:**
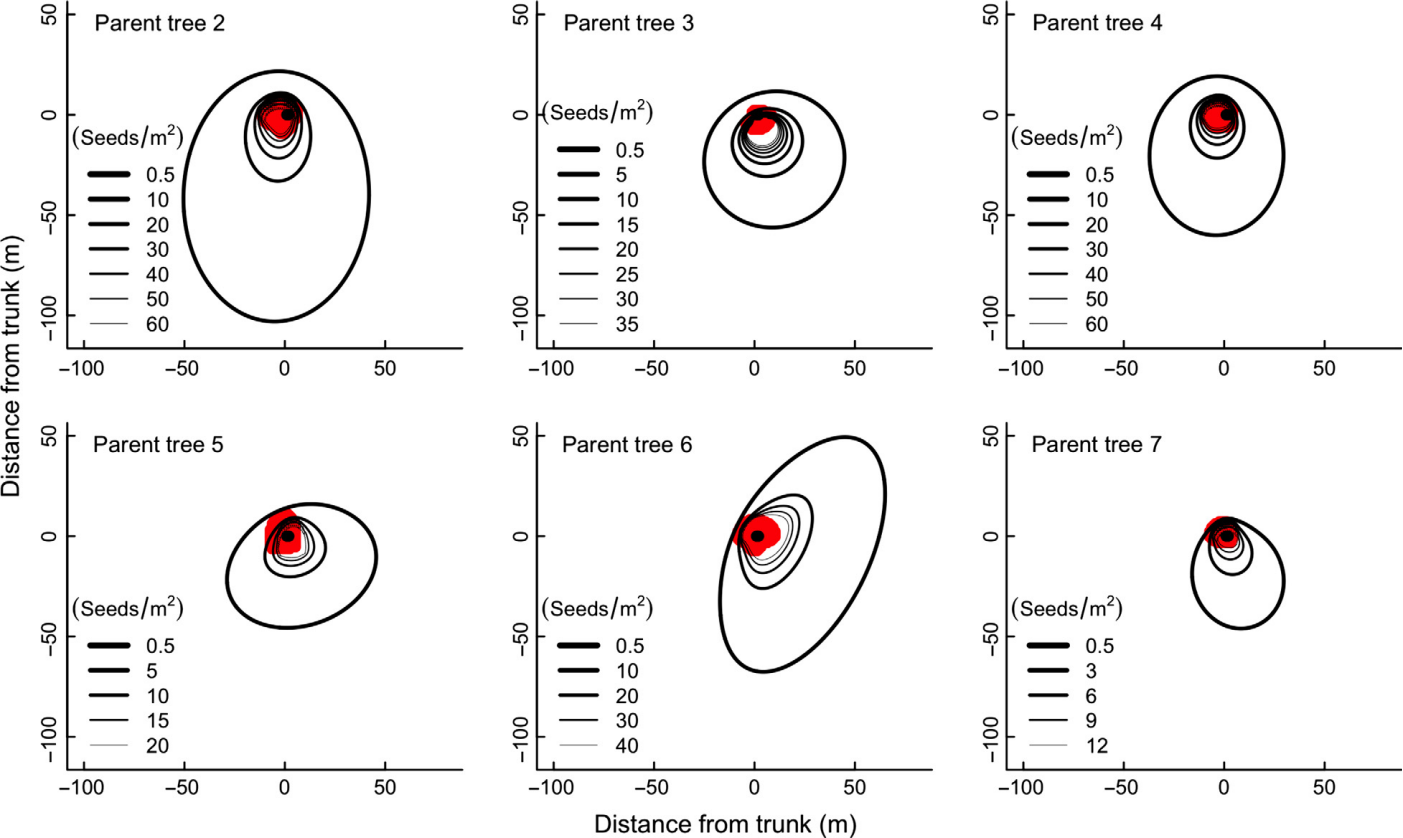
Fitted anisotropic dispersal kernels for six parent trees of *Platypodium elegans* on BCI, Panama following Van Putten et al. (2012). The red circle indicates the tree's crown area. North direction is toward the top of figures.

For all parent trees, the full elliptic distorted model of Van Putten et al. (2012) best fit the measured seed shadow data. This model allows the seed shadow to be represented by nonconcentric, nested ellipses (Fig. 5; Table S4). Mean observed distance from the parent tree was better correlated with mean simulated distance from the tree trunk (*r* = 0.704) than with mean fitted distance from a point source (calculated from the dispersal kernel) (*r* = 0.615), although neither correlation was statistically significant (Table S5).

### Compare tower versus seed shadow distributions

**Figure 6:**
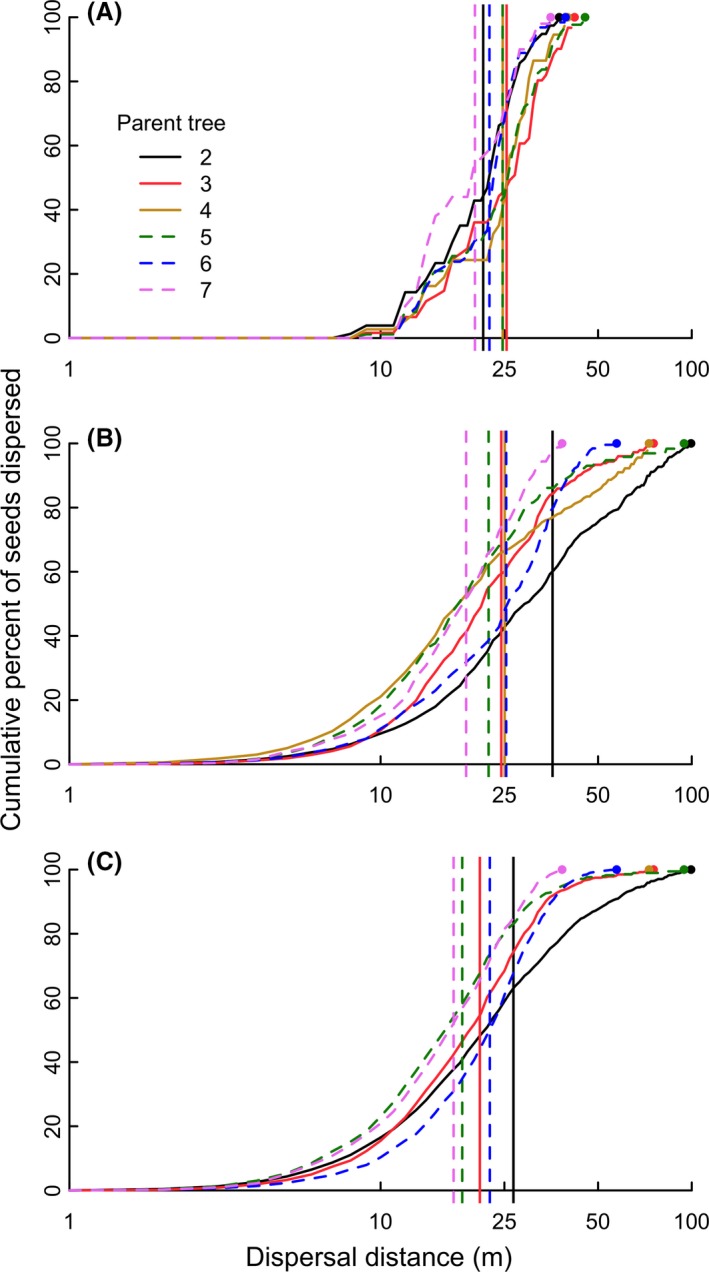
Variation in dispersal distance distributions among six parent trees for fruits of *Platypodium elegans* on BCI, Panama. (A) Fruits released from a tower (*n* = 300 each for Parents 2–5, 100 for Parents 6–7). (B) Fruits naturally dispersed in the southernmost sector of the seed shadows (*n* = 18,358, 7876, 7082, 4326, 10,234, and 2404 for Parents 2–7). (C) Fruits naturally dispersed in the entire seed shadows; see Table 3 for sample size. The maximum dispersal distance for each tree is indicated by a point (at which the cumulative distribution ends). Vertical lines show mean distances for each tree.

Rankings of the six parents in seed shadow distribution were not concordant with rankings of dispersal distances from the tower (Experiment 3) (Fig. 6, Table S6, Kendall's rank correlation coefficient τ for different percentiles: range = −0.33 to −0.90, *P *>* *0.05 for all). Further, rankings of different observed seed shadow percentiles were not consistent among parents (Fig. 6B, C; Table S6; Kendall's coefficient of concordance: M = 6, *n* = 6, W = 0.178; *X* ^2^_*r* _= 5.34, df = 4, n.s.). In contrast, rankings of percentiles of dispersal distance from the tower were significantly concordant among these same six parents (Fig. 6A; Table S6; Kendall's coefficient of concordance: M = 6, *n* = 6, W = 0.419; *X* ^2^_*r* _= 12.57, df = 4, *P* < 0.025). Differences among parents in observed dispersal distances increased with increasing percentile more than proportionally, that is, with greater fold variation at higher percentiles, but the same was not true of dispersal distances from the tower (Table S5). The 95th percentile of natural dispersal distance varied among parents between 30 and 66 m for the entire seed shadow, and between 33 and 83 m for the pie area S in the seed shadow (Table S6). Mean dispersal distance in only the pie area S in the seed shadow, as well as the entire seed shadow, differed significantly among parents (one‐way ANOVAs *P *<* *0.001) (Figs. 4 and 6B, Table S6). In contrast, mean dispersal distance from the tower did not differ significantly among the six parents (Experiment 3) (range = 22.3 to 24.2 m) (one‐way ANOVA, *P *>* *0.05; Table 4, Table S6), although it did differ significantly among all 20 parents (Experiment 2).

### Models of tree traits and seed shadow descriptors

Rate of descent and tree traits, combined in the mechanistic dispersal model, were poor predictors of observed dispersal distances in the field. Correlations between distances from the trunk predicted by the mechanistic model and those measured for each parent tree in the field were never statistically significant; only the maximum distance was marginally significant (correlation coefficient = 0.74 for the six parent trees) (Table 3). Correlations between distances from a point source within the crown predicted by the mechanistic model and those fitted phenomenologically were also not statistically significant (Table 3). The total seed shadow area predicted by the mechanistic model was marginally significantly correlated with the total measured seed shadow area (correlation coefficient = 0.73 for the six parent trees). The results for both the maximum distance and seed shadow area should be interpreted with caution considering that they are the two marginally significant correlations (r < 0.1) among thirteen tests of the mechanistic predictions of observed seed dispersal (Table 3, Fig. S2).

### Seed/Seedling/Sapling distances from parent trees

**Figure 7:**
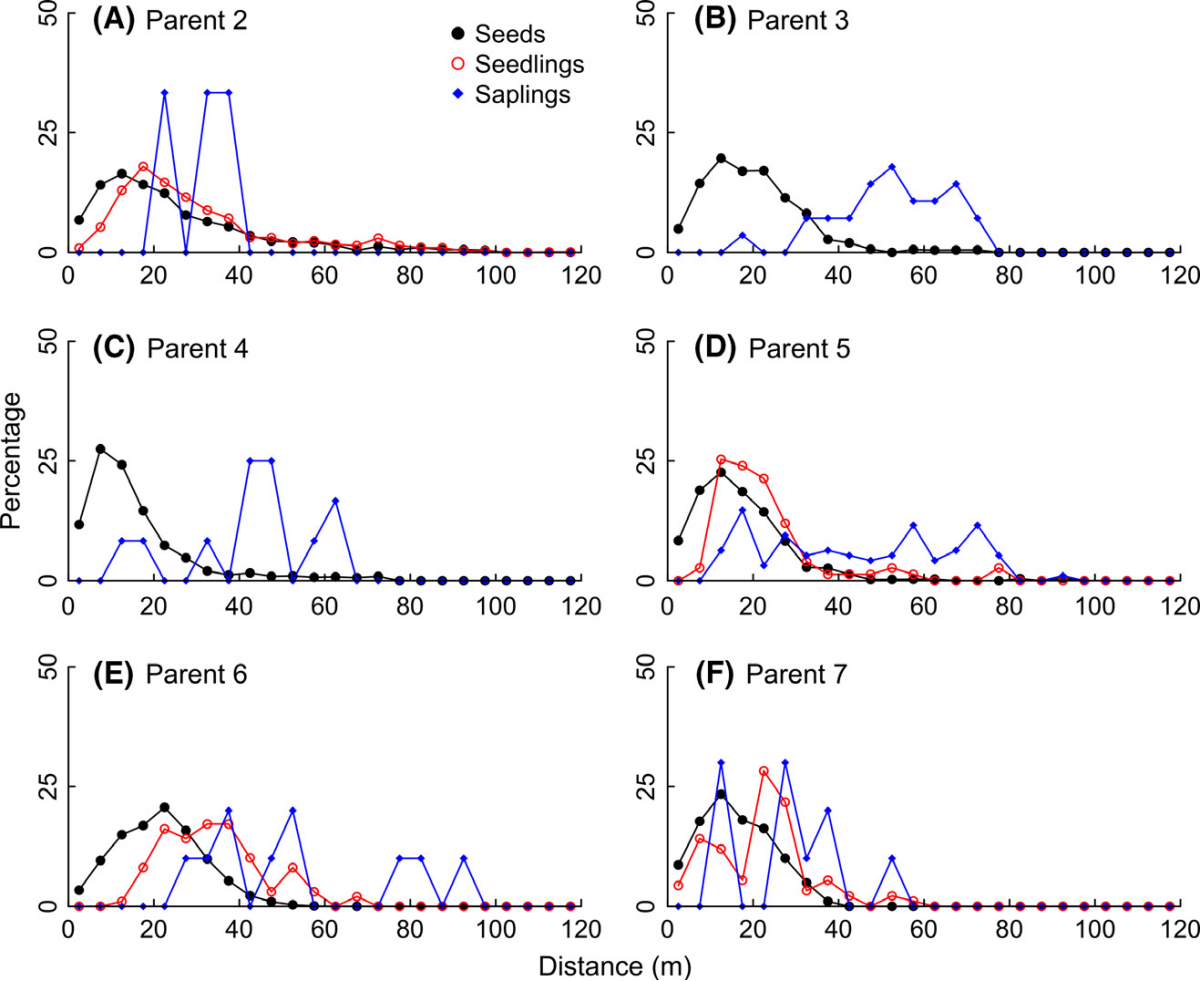
Percent of total number of offspring of *Platypodium elegans* on BCI, Panama as a function of distance from each parent tree for seeds, seedlings, and saplings. See Table 3 for sample size of seeds; seedlings: *n* = 909, 75, 99, and 92 for Parents 2, 5, 6, and 7 (no data for Parents 3 and 4); saplings: *n* = 3, 28, 12, 95, 10, and 10 for Parents 2–7.

The mean distance of offspring from the parent was greater for older than younger offspring (Fig. 7). The weighted mean distance from the parent (all parents combined) was 21.3 m (range of mean among parents = 16.1−25.4) for seeds, 31.1 m (range 21.7−52.5) for 9‐months seedlings, and 46.0 m (range 28.8−52.6) for saplings.

**Figure 8:**
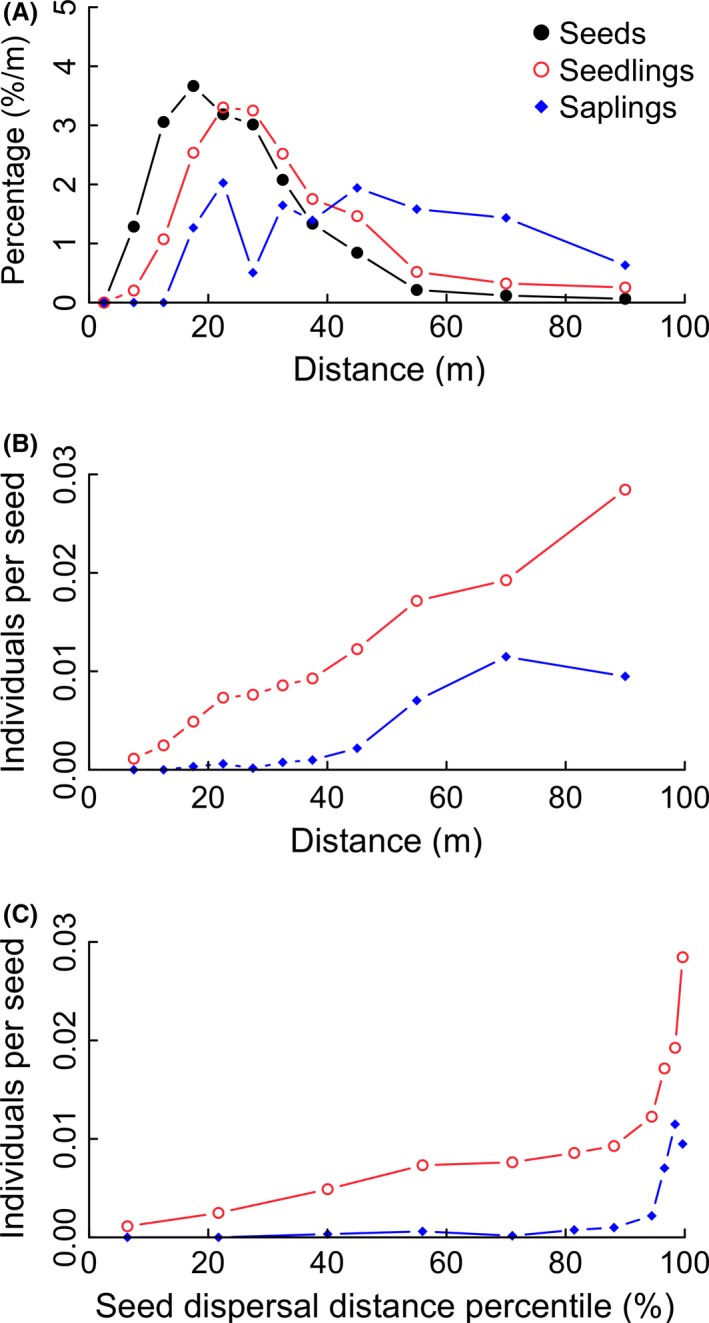
Overall distributions of seeds, seedlings, and saplings as a function of distance from parent trunk for *Platypodium elegans* on BCI, Panama. (A) Percentage of seeds (*n* = 166,226), seedlings (*n* = 1175), and saplings (*n* = 158) as a function of distance from the parent tree for all trees combined. (B) Seedlings per seed and saplings per seed as a function of distance. (C) Seedlings per seed and saplings per seed as a function of dispersal distance percentile.

No saplings occurred within the crown radius of any parent tree, despite the high seed density there (Fig. 7); indeed, 91% of all 154 saplings occurred in areas of low seed density beyond the 10 seeds per m^2^ contour of seed shadows. Only 14% of saplings were upwind (N) of the parent tree. Downwind tails of the fruit distribution represented very important areas for sapling recruitment: 46.1% (range among parents = 0–83.3%) of saplings of all parents combined occurred beyond the distance to which 95% of seeds were dispersed, that is, in the large areas of quite low density in the far tails of the distributions (Fig. 8).

## Discussion

### The limited explanatory power of fruit and tree traits for dispersal patterns

The noticeable variation in fruit mass and area among parents of *Platypodium elegans* that initiated this study became less explanatory as the study progressed from measures of rate of descent in still air to dispersal distances from the tower to natural seed shadows. Fruit traits were fairly consistent from year to year for a given parent and differed significantly among parents in a given year. Additionally, variation among parents in wing loading explained variation in rate of descent in still air. In turn, variation among parents in rate of descent explained variation in dispersal distances from the tower. However, the models explained a greater amount of variation for rate of descent than dispersal distances from the tower.

Seed shadows of parents were much more varied than tower distributions. Unlike the tower results, the tails of natural seed distributions differed more among parents than did mean and peak dispersal distances. Maximum distance, heavily impacting the shadow area, differed nearly 3‐fold among parents and was far greater than what arose from the single release from the tower. Relative to the drop of fruit in still air, the tower experiment added a single and relatively constant wind speed and direction at release, while controlling for height of release and surrounding vegetation. Under natural conditions, additional factors varied, including tree traits of crop size, crown area, height of release, and wind conditions at seed release. Thus, it is unsurprising that rankings among parents of distances from the tower and under field conditions were not in good agreement.

Fruit traits were poor predictors of natural dispersal distance. For the one parent thus investigated, distances of individual fruits were explained by wing loading^1/2^, but this model explained only 5% of the variation. Among fruit morphological traits, wing loading^1/2^, incorporating both mass and area, is the trait historically considered most predictive of dispersal capacity (Norberg 1973). The square root of wing loading strongly predicts rate of descent in still air (Green 1980), which, when coupled with height of release and mean wind speed during dispersal, predicts mean dispersal distance (Cremer 1977). However, in *Pinus halepensis,* samaras with substantial intraspecific variation in mass and wing loading^1/2^ had insignificant variation in terminal velocity (Nathan et al. 1996). Likewise, Sipe and Linnerooth (1995) found that substantial within‐tree variation in morphology weakened relationships between wing loading^1/2^ and rate of descent because wing loading^1/2^ does not take into account mass distribution and wing shape. Horn et al. (2001) found variation in rate of descent to be similar between seeds uplifted onto a building and nonlifted seeds dispersed to the ground near the parent tree.

The weak prediction by fruit traits of variation in natural dispersal distance suggests that parent tree traits may have more influence. However, tree traits, including height of release and crown area, did not explain variation in seed shadows among the six parents either. Concluding that fruit and tree traits of *Platypodium elegans* do not explain its seed dispersal based on analyses of only six trees is premature. The population‐level implications of the results are obviously limited by the sample size. In principles, higher height of release should increase dispersal distances, and indeed, tree height was found to enhance uplifting of wind‐dispersed seeds of *Jacaranda copaia* in a simulation study (Wright et al. 2008). Greater crown area should also increase the area of the seed shadow. Larger seed crops are more likely to have some seeds experiencing the important, but rare, updrafts needed for long‐distance dispersal (Nathan et al. 2002a). In general, older, larger trees have larger crop sizes and height of release, both factors that enhanced longer dispersal distances of the wind‐dispersed seeds of *Swietenia macrophylla* (Norghauer et al. 2011).

The extent of variation in fruit and tree traits compared with the extent of variation in other factors affects their ability to explain variation in dispersal distances. In this study, rate of descent differed 1.3‐fold among parents, while crown area and mean crown height differed by 2.3‐ and 1.2‐fold, respectively. The study with artificial fruits showed that fruits differing 2.0‐fold in wing loading^1/2^ had different distributions, including their tails (Augspurger and Franson 1987). However, variation in wind speed among multiple trials overrode the effect of variation in mass or area of dispersed fruits, unless differences in mass and area were quite large. Likewise, Greene and Johnson (1992) concluded that variation in terminal velocity is much smaller than variation in vertical and horizontal wind within a forest canopy and therefore contributes negligibly to variation in dispersal distance. Nathan et al. (2001) found that horizontal/vertical wind speed overrides the effects of rate of descent and height of release on dispersal distance of *Pinus halepnsis* seeds. Interspecific studies that include wider variation in fruit/seed traits than intraspecific ones may be more likely to discover that fruit/seed traits influence distributions (Guries and Nordheim 1984; Tamme et al. 2014). Muller‐Landau et al. (2008) found for nine wind‐dispersed species that a combination of rate of descent, tree height, and wind speed explained 40% of the variation in mean dispersal distance and clumping.

### The failure of most seeds to be dispersed far

Most seeds of *Platypodium elegans* were not dispersed far, consistent with other studies on wind‐dispersed trees. Clark et al. (2005) in a study of three wind‐dispersed tropical trees found that up to 65% of seed were nondispersed and only 1–2% reached distances > 60 m from the parent tree. About one‐sixth of *Platypodium elegans* fruits were nondispersed. They may not have experienced their spinning state (Burrow 1975). One model predicted modal distance of wind‐dispersed seeds to be roughly equal to canopy height (Nathan et al. 2002a). For *Platypodium elegans,* mean modal dispersal distance was 14 m, about half of canopy height. Thus, densities were high under and near the parent tree, while most of the shadow area had low densities, setting the stage for density‐dependent mortality near the parent tree and recruitment only at far distances in this species. The spatial pattern of recruitment is also affected by offspring encountering light gaps at greater dispersal distances. An experimental study with *Platypodium elegans* demonstrated seedling survival to be impacted by distance, density, and gaps (Augspurger and Kelly 1984). Any gap influence may have continued in the postseedling phase and affected the distribution of saplings.

### The important yet poorly understood seed shadow tails

The recruitment results brought sharp focus to the need to explain tails and area of seed shadows. Rare dispersal events taking seeds to far distances contributed disproportionately to successful recruitment events and enhanced parent fitness of *Platypodium elegans*. As in an earlier study (Augspurger 1983b), no saplings occurred in the area with high seed density under and near the parent. Terborgh et al. (2011), upon finding success of animal‐dispersed seeds cleaned of their pulp to far exceed that of nondispersed seeds, recommended distinguishing between dispersed and nondispersed seeds in analyzing seed distributions. All nondispersed seeds, as well as most dispersal events of *Platypodium elegans,* led to no recruitment success. As in other studies (Wada and Ribbens 1997; Jansen et al. 2008; Swamy et al. 2011), distance from parent of seedlings and particularly saplings was considerably greater than that of seeds. Norghauer et al. (2011) found the sapling to seed ratio to peak about 35–50 m downwind from parent trees of *Sweitenia macrophylla*. This focus on tails as the most successful recruitment area may not be warranted for all species. Dispersal to far distances may take a seed outside of the preferred habitat and reduce recruitment. In experimentally created fruit distributions of *Tachigalia versicolor*, recruitment was lower at extreme long distances (up to 1.8 km) with much lower density (1 per 5‐m interval) than nearer the parent, perhaps because of predator satiation at near distances with high densities (Augspurger and Kitajima 1992).

The field sampling design applied here, like most empirical sampling designs, was liable to miss rare long‐distance dispersal events. One postrelease updraft event during the artificial fruit experiment took 25–30 fruits up and away from the majority; 12 were located 172–277 m from the tower, the first being 65 m beyond the end of the measured seed shadow (Augspurger and Franson 1987). Clearly, such extreme tails were not measured adequately in this study. Bullock and Clarke (2000) recommend that the percentage of annuli sampled should be equal at all distances and sampling should be expanded to farther distances than is customarily measured. Alternatively, if long‐distance dispersal is disproportionately important for recruitment, then higher sampling intensity is needed at longer distances. In practice, however, such recommendations are rarely followed because of the great field effort required.

### Future directions and conclusions

The focus of this study on interpreting morphological variation in light of dispersal potential ignores other forces that may shape the physical traits of diaspores. The observed variation in diaspore traits may be important for other biological/ecological processes and tradeoffs, including seed predation, chemical defense, and photosynthesis. Bruchid beetles eat *Platypodium elegans* seeds and must drill through a very thick fruit wall surrounding the seed. The large wings of *Platypodium elegans* fruits are bright green and hydrated for up to a year prior to drying to brown by the time of dispersal. Photosynthetic products from the large wing may be critical for seed development and energy provisions for the embryo and developing seedling.

For intraspecific studies of dispersal, the focus should not be on morphological variation of the fruit or seed but on largely unstudied factors that enhance dispersal to greater distances. Unstudied factors include the interaction of wind and updrafts with the abscission process, temporal complexity in horizontal and vertical wind speeds, as well as updrafts after fruit release, and surrounding vegetation. Fruits and seeds may combine aerodynamic shapes and release mechanisms that cause greater abscission when the wind pulls in an upward direction (Greene and Johnson 1995; Mauer et al. 2013). Variation in wind speed, direction, and uplift occurs at the scale of individual fruits under different atmospheric conditions (Wright et al. 2008). Fruit display angle affects wind updrafts releasing seeds, and the timing of abscission affects uplift frequency and direction of winds (Mauer et al. 2013). Finally, the change in wind speed after release and collision with surrounding vegetation affects dispersal (Pounden et al. 2008).

We conclude that any study of functional traits explaining seed shadows should be coupled with an appraisal of its consequences for recruitment. Knowing that a species has a nonrandom recruitment pattern draws attention to those aspects of the shadow most important to explain. Mechanistic and modeling studies of wind dispersal need to focus on the “rare” dispersal events that contribute to longer tails (Portnoy and Willson 1993; Nathan 2006) and greater area to seed shadows rather than the majority of events that result largely in recruitment failure under and closer to the parent.

## Conflict of Interest

None declared.

## Data accessibility

R scripts for data analysis – in Supplemental Information.

Data archived at http://datadryad.org (Augspurger). doi: 10.5061/dryad.296ds

## Supporting information


**Methods S1.** Study Site, Study Species, and Focal Parent Trees.
**Methods S2.** Fruit Traits, Rate of Descent, and Dispersal Distance from Tower.
**Methods S3.** Models: Fruit Traits Predict Rate of Descent and Dispersal Distance from Tower.
**Methods S4.** Seed Shadows.
**Methods S5.** Models of Fruit and Tree Traits and Seed Shadow Descriptors.
**Methods S6.** Seed/Seedling/Sapling Distances from Parent Trees.
**Table S1.** Results of 2‐way ANOVA examining the effect of parent, year, and their interaction on variation in mass, area, and wing‐loading^1/2^ of fruits of 18 parents*Platypodium elegans* on BCI, Panama.
**Table S2.** Fruit statistics by parent and years, with tests of differences between years and among parents of *Platypodium elegans* on BCI, Panama.
**Table S3.** Percentiles of the dispersal distances of fruits released from the tower for each parent tree of *Platypodium elegans* on BCI, Panama.
**Table S4.** Fitted fecundity and dispersal parameters for the six parents of *Platypodium elegans* with censused seed shadows on BCI, Panama.
**Table S5.** Tree‐level average dispersal distances calculated using different methods.
**Table S6.** Comparisons of seed shadows resulting from dispersal from the tower with those resulting from natural dispersal from the parent tree for six parent trees of *Platypodium elegans* on BCI, Panama.
**Figure S1.** Comparison among parent trees (one line per tree) of dispersal distance distributions from the tower.
**Figure S2.** Seed shadows predicted from a mechanistic model of seed dispersal incorporating measured intraspecific variation in rate of descent and tree traits.
