# Supplementary material for: Intraspecific variation in seed dispersal of a Neotropical tree and its relationship to fruit and tree traits
Source: Ecol Evol. 2016 Jan 25;6(4):1128–42. doi: 10.1002/ece3.1905 (PMC4725333; doi:10.1002/ece3.1905)
Supplement: Supplementary file 1 — Methods S1. Study Site, Study Species, and Focal Parent Trees. Methods S2. Fruit Traits, Rate of Descent, and Dispersal Distance from Tower. Methods S3. Models: Fruit Traits Predict Rate of Descent and Dispersal Distance from Tower. Methods S4. Seed Shadows. Methods S5. Models of Fruit and Tree Traits and Seed Shadow Descriptors. Methods S6. Seed/Seedling/Sapling Distances from Parent Trees. Table S1. Results of 2‐way ANOVA examining the effect of parent, year, and their interaction on variation in mass, area, and wing‐loading1/2 of fruits of 18 parentsPlatypodium elegans on BCI, Panama. Table S2. Fruit statistics by parent and years, with tests of differences between years and among parents of Platypodium elegans on BCI, Panama. Table S3. Percentiles of the dispersal distances of fruits released from the tower for each parent tree of Platypodium elegans on BCI, Panama. Table S4. Fitted fecundity and dispersal parameters for the six parents of Platypodium elegans with censused seed shadows on BCI, Panama. Table S5. Tree‐level average dispersal distances calculated using different methods. Table S6. Comparisons of seed shadows resulting from dispersal from the tower with those resulting from natural dispersal from the parent tree for six parent trees of Platypodium elegans on BCI, Panama. Figure S1. Comparison among parent trees (one line per tree) of dispersal distance distributions from the tower. Figure S2. Seed shadows predicted from a mechanistic model of seed dispersal incorporating measured intraspecific variation in rate of descent and tree traits. [file ECE3-6-1128-s001.docx]

SUPPLEMENTAL INFORMATION FOR ARTICLE IN *ECOLOGY AND EVOLUTION*

**Intraspecific variation in seed dispersal of a Neotropical tree and its relationship to fruit and tree traits**

Carol K. Augspurger^a^, Susan E. Franson^b^, K.C. Cushman^cd^ and Helene C. Muller-Landau^c^

^a^Department of Plant Biology, University of Illinois, Urbana, IL 61801, USA

^b^EPA, 26 W. Martin Luther King Jr. Drive, Cincinnati, OH 45268, USA

^c^Smithsonian Tropical Research Institute, Apartado Postal 0843-03092, Panamá, República de Panamá

^d^Department of Ecology and Evolutionary Biology, Brown University, 80

Waterman St., Providence, RI 02912, USA

**Materials and Methods**

**Methods S1**

*Study Site, Study Species, and Focal Parent Trees*

The thick woody fruit enclosing the seed prevented distinguishing between fruits with one vs. zero seeds.

**Methods S2**

*Fruit Traits, Rate of Descent, and Dispersal Distance from Tower*

*Details on rate of descent.* The height of 27 m used in the measurements of rate of descent is roughly comparable to the mean height of a fruit on a tree of this species (mean crown height = 28.5 m; n = 6). The rate of descent (cm · sec^-1^) is not precisely equivalent to terminal velocity because the fruit requires about 1.5 sec (8% of total time) after release before it begins aerodynamic movement and reaches terminal velocity. Expected standard dispersal distance was calculated from the rate of descent as (standard dispersal distance) = (height of release)*(wind speed)/mean rate of descent) = 40/(mean rate of descent)(Cremer 1977). The value of wind speed (1 m/s) was selected because it is a round number close to the observed mean effective wind speed during dispersal measured in a previous study when seeds were released under above-canopy wind speeds of 7 m·sec^-1^ (the same wind speed as in this study) (Augspurger and Franson 1987). The height of 40 m was chosen because it corresponds to the height of the tower in the tower dispersal experiment.

*Details on the tower dispersal experiment.* In the tower dispersal experiments, each recovered fruit was assigned the Cartesian coordinates of the center of the 2.5 x 2.5 m quadrat in which it was recovered, and the distance between that point and the SW side of the tower. Overall, 94% of the fruits were recovered.

**Methods S3**

*Models: Fruit Traits Predict Rate of Descent and Dispersal Distance from Tower*

The following models were used to predict the rate of descent of seeds. Abbreviations used in the following table are:

A = Fruit area (cm^2^)

RD = Rate of descent (m/s)

M = Fruit mass (g)

WL = Wing-loading (mg/cm^2^)

b_i_ = fitted model parameter i

I = fitted model intercept

All models were fit by linear regression to log-transformed data of both predictors and response variables.

Descent rate models:

| Predicted quantity | Models |
| --- | --- |
| Log rate of descent of individual fruits from Year 2 as a function of log wing-loading, area, and/or mass | 1. Log(RD) ~ I |
|  | 2. Log(RD) ~ I + 0.5 Log(WL) |
|  | 3. Log(RD) ~ I + b_1_ Log(WL) |
|  | 4. Log(RD) ~ I + b_1_ Log(A) |
|  | 5. Log(RD) ~ I + b_1_ Log(M) |
|  | 6. Log(RD) ~ I + b_1_ Log(A) + b_2_ Log(M) |

Dispersal distance from tower models:

| Predicted quantity | Models |
| --- | --- |
| Log dispersal distance of individual fruits from Year 2 as a function of log wing-loading, area, mass, and/or rate of descent | 1. Log(Distance) ~ I |
|  | 2. Log(Distance) ~ I + 0.5 Log(WL) |
|  | 3. Log(Distance) ~ I + b_1_ Log(WL) |
|  | 4. Log(Distance) ~ I + b_1_ Log(A) |
|  | 5. Log(Distance) ~ I + b_1_ Log(M) |
|  | 6. Log(Distance) ~ I + b_1_ Log(A) + b_2_ Log(M) |
|  | 7. Log(Distance) ~ I – 1*Log(RD) |
|  | 8. Log(Distance) ~ I + b_1_ Log(RD) |
|  | 9 Log(Distance) ~ I + b_1_ Log(WL) + b_2_ Log(RD) |
|  | 10. Log(Distance) ~ I + b_1_ Log(A) + b_2_ Log(RD) |
|  | 11. Log(Distance) ~ I + b_1_ Log(M) + b_2_ Log(RD) |
|  | 12. Log(Distance) ~ I + b_1_ Log(M) + b_2_ Log(A) + b_3_ Log(RD) |

When applying fruit-level relationships to tree-level data, we used corresponding means and percentiles of predicted rates (e.g., 75^th^ percentile of fruit traits to predict 75^th^ percentile of descent rates). For each quantity, we used fitted values for the single best fruit-level model (lowest AIC) and for the expected best models from first principles (wing-loading^1/2^ for descent rate, wing-loading^1/2^ or descent rate^-1^ for dispersal distance).

**Methods S4**

*Seed Shadows*

*Sampling details.* Sampling was more intensive to the south because winds blow from the N or NE in the dry season.  For the two cardinal directions not sampled directly (NE and NW), fruit densities were estimated as averages of fruit densities in the two adjacent transects.

*Calculating crop size and mean dispersal distance.* We define $\rho_{ij}$ as the number of seeds per m^2^ (density) where *i* is an index for the number of the quadrat from the tree (m) and $j \in\{1,3,4,5,6,7\}$ is a sampled transect. We define *r_i_* as the distance of the *i*th quadrat from the center of the trunk, equal to the sum of *i* and half the diameter of the trunk. (That is, $\rho_{i2}=(\rho_{i1}+ \rho_{i3})/2$ and $\rho_{i8}=(\rho_{i1}+ \rho_{i7})/2$, where *j*=2 denotes NE and *j*=8 denotes NW.) We estimated the numbers of seeds in the 1/8^th^ of the full circle centered on each transect, *j*, at each distance *i*, by assuming that the seed density on the transect, $\rho_{ij}$, was representative of that in the entire fractional arc. We estimated the total crop size for the tree as

$$\mathcal{N=}\sum_{i=1}^{m} \sum_{j=1}^{8} \rho_{ij}\pi\frac{\left( r_{i}+0.5 \right)^{2}- \left( r_{i}-0.5 \right)^{2}}{8}$$

where *m* is the maximal seed dispersal distances on any transect. Similarly we estimated the overall mean dispersal distance as:

$X= \frac{1}{\mathcal{N}}\sum_{i=1}^{m} \sum_{j=1}^{8} r_{i}\rho_{ij}\pi\frac{\left( r_{i}+0.5 \right)^{2}- \left( r_{i}-0.5 \right)^{2}}{8}$

*Making contour maps.* To generate contour maps with isopleths of seed density at various intervals around the parent tree, the perimeter of the seed shadow was determined using the maximum dispersal distance of each transect and adding a curved line to connect those eight points. A suitable contour interval was selected by preliminary inspection of the data. Successive contour lines were drawn from the perimeter by proceeding inward along each transect until an increase in seed density appropriate to the chosen contour interval was reached, and the points were connected as above. When the sample density was highly variable over a short interval of transect, 3- or 5- point running means were calculated to smooth the curves.

*Fitting seed shadows.* When fitting the model of Van Putten et al. (2012), we assumed that the parent tree was the only source, and we treated the entire area of the crown as a source. To this effect, instead of fitting the model with one point seed source at the trunk, we modeled it as a grid of seed sources located at an even spacing of 0.5 m throughout the crown area. The crown area was defined for each sector between measured crown radii as the ellipse centered at the trunk passing through the measured crown coordinates; when no such ellipse existed, a straight line through the measured crown coordinates was used instead to define the crown area (this occurred in only two sectors). We treated all 1-m^2^ quadrats with measured seed densities as 1-m^2^ seed traps with coordinates at the center of the quadrat, plus half the diameter of the trunk. For each transect, we added an additional 50 quadrats with an assumed seed count of zero (20 of these were actually observed to be free of seeds; further quadrats were added as zero in the fits to better constrain the model).

The alternative dispersal kernels fitted were the full suite described in Van Putten et al. (2012), ranging from a simple 1-parameter isotropic kernel, to complex anisotropic models with separate terms for rotation of the kernel, coherency (flattening of contour lines along their common axis), drift (pulling of centers of contour lines along their common axis), and shift (displacement of the center of the kernel from the source). For each increasingly complex model, initial values were estimated from the results of fitting the previous model of lower complexity. The fits were done using the code provided by Van Putten et al. (2012).

*Compare Tower vs. Seed Shadow Distributions.* Because tower distributions were affected by only one major wind event blowing S, while seed shadows were created by multiple wind events blowing predominantly, but not exclusively, to the S, we did two sets of comparisons of distributions of fruits from the tower vs. around the parent tree: first based on only the 1/8 pie-shaped area to the S of each seed shadow, and second based on the entire seed shadow.

**Methods S5**

*Models of Fruit and Tree Traits and Seed Shadow Descriptors*

*Tree Traits.* To quantify tree traits, two individuals independently measured DBH, maximum tree height (height of tallest branch using range finder), minimum crown height (height of first branching point using range finder), and crown radii (tape measured from edge of trunk to observed edge of crown) in the six directions of counted fruits; mean values of the two measurements were used (Table 1). From these we calculated mean crown height as the average of maximum and minimum crown heights, and crown area from the geometric mean of crown radii.

*The model.* The mechanistic seed dispersal model WINDISPER-E was used to predict seed shadows from fruit and tree traits (Nathan et al. 2002). WINDISPER-E is a modified ballistic model:

$$D=\frac{U_{c}\cdot H_{c}}{\alpha\left( F-W \right)}\left( exp\left( \frac{\alpha\left( H-H_{c} \right)}{H_{c}} \right)-exp(-\alpha) \right)$$

where *D* is dispersal distance (m), *U_c_* is horizontal windspeed (ms^-1^), *H_c_* is the height of the canopy top (m), α is as attenuation coefficient (unitless), *F* is the terminal velocity of seeds (ms^-1^), *W* is the vertical windspeed (ms^-1^), and *H* is the height of seed release (m). For each tree, we simulated the dispersal distance of 50,000 seeds using the following model inputs:

*U_c_* : mean horizontal windspeed sampled from eddy flux data for each seed

*H_c_* : 22 m (constant for all seeds and trees, mean canopy height for BCI (Mascaro et al. 2011)

α : 3 (constant for all seeds and trees)

*F* : descent rate in still air sampled for each seed from measurements for the parent tree

*W* : 0 ms^-1^ (constant for all seeds and trees)

*H* : sampled from the range between the minimum and maximum measured crown height

for the parent tree

For each seed, a single descent rate was sampled from the measured descent rates from its respective parent tree. We also sampled a starting time from within instantaneous measurements of wind speed and direction (10 Hz measurements from an eddy flux tower on Barro Colorado Island) from 10 days of daytime measurements (08:00-18:00) in 2015 (January 10, January 20, January 30, February 9, February 19, March 1, March 11, March 21, March 31, April 24). The selected days span the dry season when most seeds are dispersed (H. Muller-Landau and S. Wright, unpublished data). Horizontal wind speed and wind direction were averaged over the estimated period that the seed spends falling (tree height/descent rate). For each seed, a starting position was sampled from within the measured crown area and an ending position was calculated using the modeled dispersal distance and mean wind direction.

*Correlations of predicted distance, measured distance, and dispersal kernel values.* Pearson correlations were calculated between seed dispersal distance from the trunk (mechanistic model) and measured dispersal distance from the trunk, predicted seed dispersal distance from the point of release within the canopy (mechanistic model) and fitted dispersal distance from the point of release (dispersal kernel), and total predicted seed shadow area (mechanistic model) and area measured around each parent tree.

**Methods S6**

*Seed/Seedling/Sapling Distances from Parent Trees.* If no other parent’s seed shadow area was overlapping, the sampled areas were extended up to 30 m beyond the seed shadow of parents, assuming that dispersal distances may have been greater than in the year of mapping seed shadows. For each sapling, distance to tree trunk of the parent was measured directly; for each seedling, it was estimated to the nearest 5 m by pacing from lain transects.

**References**

Augspurger, C.K. & Franson, S.E. (1987) Wind dispersal of artificial fruits varying in mass,

area, and morphology. *Ecology,* **68**, 27-42.

Cremer, K.W. (1977) Distance of seed dispersal in eucalypts estimated from seed weights.

*Australian Forest Research,* **7**, 225-228.

Mascaro, J. Asner, G.P, Muller-Landau, H.C. van Breugel, M, Hall, J, & Dahlin, K. (2011)

Controls over aboveground forest carbon density on Barro Colorado Island, Panama. Biogeosciences, **8**, 1615-1629.

Nathan, R. Horn, H.S., Chave, J., and Levin, S.A. (2002) Mechanistic models for tree seed

dispersal by wind in dense forests and open landscapes. Seed Dispersal and Frugivory.

Ecology, Evolution and Conservation (eds D.J. Levey, Silva, W.R. & Galetti, M.), pp. 69-82. CAB International, Wallingford, UK.

Van Putten, B., Visser, M.D., Muller-Landau, H.C., & Jansen, P.A. (2012) Distorted-distance

models for directional dispersal: a general framework with application to a wind-dispersed tree. *Methods in Ecology and Evolution,* **3**, 642–652.

Table S1. Results of 2-way ANOVA examining the effect of parent, year, and their interaction on variation in mass, area, and wing-loading^1/2^ of fruits of 18 parents *Platypodium elegans* on BCI, Panama.

| MASS | SS | df | MS | F | *p* |
| --- | --- | --- | --- | --- | --- |
| Parent | 377.185 | 17 | 22.187 | 37.436 | 0.001 |
| Year | 2.735 | 1 | 2.735 | 4.614 | 0.032 |
| Interaction | 56.694 | 17 | 3.335 | 5.627 | 0.001 |
| Error | 1903.071 | 3211 | 0.593 |  |  |
| Total | 2338.321 | 3246 | 0.720 |  |  |
|  |  |  |  |  |  |
| AREA |  |  |  |  |  |
| Parent | 30177.657 | 17 | 1775.156 | 238.698 | 0.001 |
| Year | 675.147 | 1 | 675.147 | 90.784 | 0.001 |
| Interaction | 2097.358 | 17 | 123.374 | 16.590 | 0.001 |
| Error | 18770.542 | 2524 | 7.437 |  |  |
| Total | 52078.570 | 2559 | 20.351 |  |  |
|  |  |  |  |  |  |
| WING-LOADING^1/2^ |  |  |  |  |  |
| Parent | 1113488.685 | 17 | 65499.334 | 57.260 | 0.001 |
| Year | 829.731 | 1 | 829.731 | 0.725 | 0.395 |
| Interaction | 152029.512 | 17 | 8942.912 | 7.818 | 0.001 |
| Error | 2887173.172 | 2524 | 1143.888 |  |  |
| Total | 4153284.871 | 2559 | 1623.011 |  |  |

Table S2. Fruit statistics by parent and years, with tests of differences between years and among parents of *Platypodium elegans* on BCI, Panama. Unless otherwise noted, values are means (with standard errors). Bold indicates a significant difference between years for a given parent and trait (*t-*test, *p*<0.05). Within each column, different small letters indicate significant differences among parents (1-way ANOVA, then SNK with Bonferroni corrections at *p*<0. 05). Rate of descent was measured with a 27-m drop in still air. Expected standard dispersal distance was calculated as (40 m Height) * (1 m s^-1^ Windspeed)/(Rate of Descent). Actual mean and maximum distances are observed dispersal from experimental release from a 40-m tower. N = total number for all fruits of all parents combined. Asterisks (*) denote parents for which seed shadows were also measured.

| Parent | Fruit Mass (g)    Yr 1 Yr 2 | | Fruit Area (cm^2^)    Yr 1 Yr 2 | | Fruit Wing-loading^1/2^  (g^1/2^ cm^-1^)  Yr 1 Yr 2 | | Rate of  Descent (m s^-1^)  Yr 2 | Expected  Standard  Distance  (m)  Yr 2 | Actual  Mean  Distance  (m)  Yr 2 | Actual Max.  Distance  (m)  Yr 2 |
| --- | --- | --- | --- | --- | --- | --- | --- | --- | --- | --- |
| 1 | **1.6d**  **(0.03)** | **1.4ab**  **(0.02)** | 21.6d  (0.6) | 20.6cd  (0.2) | **259.1bc**  **(6.9)** | **245.9a**  **(1.7)** | 1.8a  (0.02) | 22.9gh  (0.3) | 24.5abc  (0.9) | 70.0 |
| 2* | **2.3i**  **(0.03)** | **1.7bcd**  **(0.03)** | **28.2g**  **(0.2)** | **25.1f**  **(0.3)** | **272.1bcd**  **(1.6)** | **252.7a**  **(1.7)** | 1.9abc  (0.07) | 22.3gh  (0.4) | 25.1abc  (0.9) | 45.5 |
| 3* | 2.2i  (0.02) | 2.3def  (0.2) | **27.4g**  **(0.3)** | **23.9e**  **(0.3)** | **275.0cde**  **(1.3)** | **289.0def**  **(6.2)** | 2.1bcd  (0.06) | 19.6cde  (0.4) | 22.7abc  (0.9) | 45.0 |
| 4* | **1.5bc**  **(0.02)** | **1.6abc**  **(0.03)** | **23.6d**  **(0.2)** | **22.5e**  **(0.4)** | **240.2a**  **(1.9)** | **257.0ab**  **(2.8)** | 1.9abc  (0.04) | 21.7fgh  (0.4) | 25.7abc  (1.3) | 45.0 |
| 5* | **1.9ef**  **(0.02)** | **1.6abc**  **(0.04)** | 24.9ef  (0.3) | 24.9f  (0.3) | **268.2bcd**  **(1.8)** | **243.5a**  **(2.4)** | 1.8ab  (0.06) | 22.9h  (0.4) | 26.0bc  (1.1) | 44.0 |
| 6* | **1.2a**  **(0.02)** | **1.5abc**  **(0.03)** | **16.3a**  **(0.2)** | **17.9b**  **(0.3)** | **257.8b**  **(1.8)** | **275.8bcd**  **(2.6)** | 2.1abcd  (0.08) | 20.8ef  (0.5) | 22.3abc  (0.8) | 39.5 |
| 7* | **2.3i**  **(0.03)** | **2.4def**  **(0.03)** | **31.3h**  **(0.9)** | **28.8h**  **(0.4)** | **259.6bc**  **(4.3)** | **275.3bcd**  **(2.1)** | 2.0abcd  (0.04) | 19.9cdef  (0.3) | 19.9a  (1.0) | 35.5 |
| 8 | **1.6cd**  **(0.03)** | **2.0cde**  **(0.1)** | **17.3ab**  **(0.5)** | **20.6cd**  **(0.3)** | 281.8cde  (5.0) | 293.3defg  (7.9) | 2.1abcd  (0.04) | 19.5cde  (0.4) | 24.9abc  (0.9) | 36.5 |
| 9 | **1.4b**  **(0.02)** | **1.2a**  **(0.03)** | 18.4abc  (0.5) | 17.9b  (0.2) | **272.8bcde**  **(7.4)** | **248.4a**  **(2.2)** | 1.9abc  (0.03) | 21.3fg  (0.3) | 22.9abc  (0.9) | 42.5 |
| 10 | **2.0fg**  **(0.03)** | **1.7abc**  **(0.02)** | **25.1ef**  **(0.4)** | **23.7e**  **(0.3)** | **273.3cde**  **(2.6)** | **254.3a**  **(1.7)** | 1.9abc  (0.02) | 21.2fg  (0.3) | 24.6abc  (0.8) | 50.0 |
| 11 | **1.6d**  **(0.02)** | **1.5abc**  **(0.02)** | 18.2ab  (0.2) | 18.7b  (0.2) | **284.6de**  **(2.5)** | **272.3bcd**  **(2.2)** | 2.0abc  (0.03) | 20.6def  (0.3) | 26.1bc  (1.3) | 73.5 |
| 12 | **2.0g**  **(0.03)** | **1.9cd**  **(0.02)** | **23.8de**  **(0.9)** | **21.2d**  **(0.2)** | 279.6cde  (7.7) | 287.0cdef  (1.5) | 2.2cd  (0.03) | 18.5abc  (0.2) | 21.3ab  (0.8) | 45.0 |
| 13 | 1.9ef  (0.03) | 1.9cde  (0.02) | **21.8d**  **(0.3)** | **22.8e**  **(0.3)** | 287.0de  (7.9) | 280.8cde  (1.9) | 2.0abcd  (0.09) | 20.4def  (0.3) | 24.3abc  (0.8) | 36.0 |
| 14 | **2.3i**  **(0.03)** | **1.7abcd**  **(0.05)** | **26.8g**  **(0.2)** | **22.8e**  **(0.5)** | **278.3cde**  **(1.8)** | **264.4abc**  **(2.9)** | 1.9abc  (0.04) | 21.9fgh  (0.4) | 27.4c  (1.9) | 73.5 |
| 15 | 2.2i  (0.02) | 2.2def  (0.2) | 19.5bc  (0.2) | 19.7c  (0.6) | 323.2g  (1.6) | 318.8h  (7.6) | 2.0abc  (0.02) | 20.2def  (0.2) | 23.3abc  (0.8) | 45.0 |
| 16 | 2.1h  (0.03) | 2.3def  (0.2) | **28.3g**  **(0.7)** | **23.8e**  **(0.3)** | **265.6bcd**  **(3.9)** | **289.4def**  **(7.2)** | 2.2cd  (0.09) | 19.4cde  (0.4) | 22.8abc  (0.8) | 45.0 |
| 17 | 1.6cd  (0.02) | 1.5abc  (0.02 | **18.6bc**  **(0.2)** | **15.4a**  **(0.3)** | **279.8cde**  **(2.0)** | **304.1fgh**  **(2.6)** | 2.3d  (0.19) | 18.6abc  (0.5) | 25.3abc  (1.3) | 73.5 |
| 18 | 2.3i  (0.02) | 2.6f  (0.3) | **22.4d**  **(0.2)** | **23.8e**  **(0.3)** | 306.4f  (1.5) | 308.8gh  (8.3) | 2.3d  (0.06) | 17.8a  (0.2) | 21.6abc  (1.1) | 73.5 |
| 19 | N/A | 2.4def  (0.04) | N/A | 26.4g  (0.2) | N/A | 286.7cdef  (2.2) | 2.2cd  (0.09) | 19.2bcd  (0.3) | 25.5abc  (1.0) | 45.0 |
| 20 | N/A | 2.4ef  (0.02) | N/A | 24.9f  (0.3) | N/A | 298.4efg  (1.9) | 2.4d  (0.12) | 17.8ab  (0.3) | 23.2abc  (1.0) | 45.0 |
| Overall | 1.9  (0.01) | 1.9  (0.03) | 23.2  (0.1) | 22.4  (0.1) | 277.3  (0.8) | 277.8  (1.1) | 2.0  (0.02) | 20.3  (0.3) | 23.8  (0.2) | 73.5 |
| N | 1997 | 1629 | 1134 | 1625 | 1134 | 1625 | 1478 | 1478 | 1301 |  |

Table S3. Percentiles of the dispersal distances of fruits released from the tower for each parent tree of *Platypodium elegans* on BCI, Panama.

|  | Dispersal Distance Percentiles (m) | | | | | |
| --- | --- | --- | --- | --- | --- | --- |
| Parent | 25% | 50% | 75% | 90% | 95% | 100% |
| 1 | 17.0 | 25.0 | 29.0 | 32.5 | 35.5 | 70.0 |
| 2 | 19.0 | 24.0 | 25.5 | 30.5 | 32.0 | 39.5 |
| 3 | 16.5 | 22.5 | 26.5 | 33.0 | 37.0 | 45.0 |
| 4 | 22.5 | 25.5 | 30.0 | 35.0 | 39.5 | 45.0 |
| 5 | 18.0 | 26.5 | 31.5 | 38.5 | 40.0 | 44.0 |
| 6 | 19.0 | 25.5 | 31.5 | 35.5 | 39.0 | 45.5 |
| 7 | 14.0 | 19.5 | 25.0 | 27.5 | 31.0 | 35.5 |
| 8 | 22.0 | 25.0 | 30.5 | 33.0 | 35.5 | 36.5 |
| 9 | 16.5 | 24.0 | 26.5 | 32.0 | 35.5 | 42.5 |
| 10 | 22.0 | 25.0 | 29.0 | 33.0 | 34.5 | 50.0 |
| 11 | 20.5 | 25.0 | 31.0 | 36.0 | 39.0 | 73.5 |
| 12 | 14.5 | 22.5 | 25.5 | 28.5 | 30.5 | 42.5 |
| 13 | 20.0 | 25.0 | 27.5 | 33.0 | 34.0 | 36.0 |
| 14 | 22.0 | 25.5 | 31.0 | 39.5 | 40.0 | 73.5 |
| 15 | 19.5 | 23.0 | 25.5 | 32.0 | 35.5 | 45.0 |
| 16 | 17.0 | 24.0 | 26.5 | 30.5 | 34.5 | 45.0 |
| 17 | 20.5 | 24.0 | 27.0 | 34.5 | 42.5 | 73.5 |
| 18 | 16.5 | 21.5 | 25.0 | 28.0 | 34.0 | 73.5 |
| 19 | 22.0 | 25.0 | 30.5 | 35.0 | 40.0 | 45.0 |
| 20 | 16.5 | 24.5 | 27.0 | 34.5 | 42.0 | 45.0 |

Table S4. Fitted fecundity and dispersal parameters for the six parents of *Platypodium elegans* with censused seed shadows on BCI, Panama. In all cases the best-fit dispersal kernel was the full elliptic distorted model described in van Putten et al. (2012).

| Parent | Fecundity | Distance | Coherency | Drift | Rotation | Center (x) | Center (y) | Clumping |
| --- | --- | --- | --- | --- | --- | --- | --- | --- |
|  | α | λ | β | γ | ψ | Δx | Δy | κ |
| 2 | 5.293 | 15.878 | 0.950 | 1.008 | -1.603 | 0.802 | -1.007 | 7.153 |
| 3 | 5.410 | 11.118 | 0.817 | 0.623 | -1.186 | -1.313 | 4.189 | 4.173 |
| 4 | 5.123 | 10.029 | 0.969 | 0.717 | -1.594 | 1.324 | -1.254 | 6.292 |
| 5 | 4.210 | 15.785 | 0.596 | 0.695 | -1.229 | -4.184 | 2.955 | 6.978 |
| 6 | 5.520 | 17.795 | 0.300 | 1.297 | -0.459 | -2.073 | -0.636 | 1.419 |
| 7 | 3.779 | 12.270 | 0.197 | 5.644 | -1.308 | -1.956 | -0.241 | 2.799 |

Table S5. Tree-level average dispersal distances calculated using different methods. For each parent tree of *Platypodium elegans*, dispersal distances were determined using the average distance to the trunk from fruits collected in the field on BCI, Panama (Observed distance from trunk). Average distance was also determined from fitted seed dispersal kernels using both the simulated distance from the trunk (Modeled distance from trunk) and the simulated distance from a point source within the crown (Modeled distance from point source). The Pearson correlations for values calculated with different methods were 0.704 (Observed distance from trunk and Modeled distance from trunk), 0.615 (Observed distance from trunk and Modeled distance from point source), and 0.430 (Modeled distance from trunk and Modeled distance from point source); however, no correlations were significant.

| Parent | Observed distance from trunk (m) | Modeled distance from trunk (m) | Modeled distance from point source (m) |
| --- | --- | --- | --- |
| 2 | 25.4 | 27.5 | 15.6 |
| 3 | 20.4 | 19.0 | 11.3 |
| 4 | 16.3 | 15.4 | 10.2 |
| 5 | 17.7 | 19.1 | 15.3 |
| 6 | 22 | 19.5 | 17.9 |
| 7 | 16.5 | 22.1 | 12.1 |

Table S6. Comparisons of seed shadows resulting from dispersal from the tower with those resulting from natural dispersal from the parent tree for six parent trees of *Platypodium elegans* on BCI, Panama. The main values given for the seed shadow include only the 1/8 ‘pie’ area of the seed shadow to the South, with values for the entire seed shadow in parentheses. Values under Dispersal Distance Percentiles refer to distance (m) to which a given cumulative % of all seeds were dispersed. Mean distances that differed significantly among parents (1- way ANOVA, followed by SNK, p<0.05) have different small letters. Values for Mean Rank Among Cumulative Percentages were based on ranking parents for distance at each cumulative percentage and then calculating the mean of these ranks.

| Parent-Source | Dispersal Distance Percentiles (m)  25% 50% 75% 90% 95% 100% | | | | | | Mean  Distance  (m) | Rank of  Mean Distance | Mean Rank Among  Cumulative Percentages |
| --- | --- | --- | --- | --- | --- | --- | --- | --- | --- |
| 2-Tower | 17 | 21 | 25 | 29 | 33 | 67 | 22.3^a^ | 1 | 2.7 |
| 3-Tower | 18 | 22 | 26 | 29 | 33 | 54 | 23.1^a^ | 4 | 3.1 |
| 4-Tower | 18 | 20 | 25 | 28 | 31 | 50 | 22.3^a^ | 1 | 1.9 |
| 5-Tower | 19 | 23 | 26 | 33 | 35 | 45 | 24.2^a^ | 6 | 4.7 |
| 6-Tower | 18 | 22 | 25 | 29 | 33 | 67 | 23.0^a^ | 3 | 3.6 |
| 7-Tower | 18 | 22 | 26 | 34 | 35 | 56 | 23.5^a^ | 5 | 5.0 |
| 2-Seed  Shadow | 17  (11) | 29  (19) | 49  (33) | 72  (52) | 83  (66) | 99  (99) | 35.7^e^  (25.4^d^) | 6  (6) | 5.6  (5.5) |
| 3-Seed  Shadow | 13  (11) | 20  (18) | 31  (26) | 43  (33) | 56  (41) | 75  (75) | 24.3^c^  (20.4^b^) | 3  (4) | 4.0  (4.0) |
| 4-Seed  Shadow | 11  (7) | 18  (12) | 34  (18) | 57  (30) | 66  (46) | 72  (72) | 25.0^d^  (16.3^a^) | 4  (1) | 3.1  (2.3) |
| 5-Seed  Shadow | 11  (10) | 17  (15) | 27  (22) | 40  (29) | 57  (36) | 94  (94) | 22.1^b^  (17.7^a^) | 2  (3) | 3.1  (2.7) |
| 6-Seed  Shadow | 15  (14) | 25  (20) | 33  (27) | 39  (34) | 43  (38) | 57  (57) | 25.2^d^  (22.0^c^) | 5  (5) | 3.1  (4.5) |
| 7-Seed  Shadow | 12  (9) | 18  (15) | 24  (22) | 30  (28) | 33  (30) | 38  (38) | 18.7^a^  (16.5^a^) | 1  (1) | 2.0  (1.5) |

Figure S1. Comparison among parent trees (one line per tree) of dispersal distance distributions from the tower. Each line stops at a dot, the maximum measured distance for each tree.


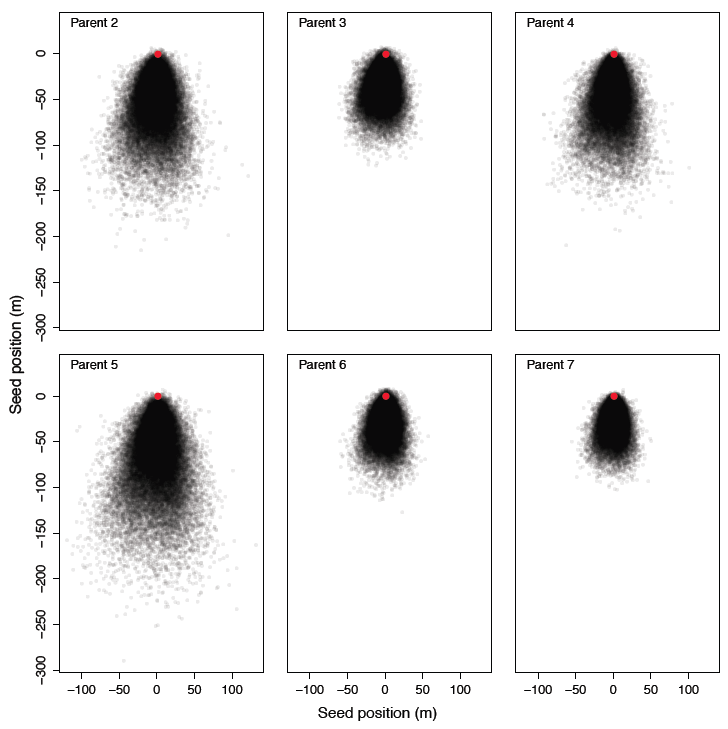


Figure S2. Seed shadows predicted from a mechanistic model of seed dispersal incorporating measured intraspecific variation in rate of descent and tree traits. For each parent tree, dispersal position was simulated for 50,000 seeds and the trunk is shown in red.

**R script 1 to make Figures**

###### Intraspecific variation in seed dispersal of a Neotropical tree and its relationship to

###### fruit and tree traits.

###### Augspurger et al.

###### Make figures.R ######

#### This file contains the code to make Figures 2,3,6,7,8,S1, and S2.

#### Figure 5 is made using modified code from van Putten et al. (2012).

#### Figure 2 ####

#### Within- and among tree variation in fruit traits, rate of descent, and dispersal distance

#### from the tower, and their interrelationships

# Load data

fruits <- read.csv('Individual fruits.csv')

# Calculate additional values for each fruit

#Square root of wingloading

fruits$SqrtWL = sqrt(fruits$Wingloading)

#Inverse of square root of wingloading

fruits$InvSqrtWL = 1/fruits$SqrtWL

#Standard dispersal distance (from a 40-m tower, assuming a 1 m/s windspeed)

fruits$StdDist = 40/(fruits$DescentRate)*1

#Find mean and standard deviation for each tree for each fruit trait

fruit.means=aggregate.data.frame(fruits[fruits$Year==1983,

c('Mass','Area','Wingloading','DescentRate','DistFrmTower','SqrtWL','InvSqrtWL','StdDist')],

by=list(fruits[fruits$Year==1983,'Parent']),FUN='mean',na.rm=T)

colnames(fruit.means)[1]='Parent'

fruit.stdev=aggregate.data.frame(fruits[fruits$Year==1983,

c('Mass','Area','Wingloading','DescentRate','DistFrmTower','SqrtWL','InvSqrtWL','StdDist')],

by=list(fruits[fruits$Year==1983,'Parent']),FUN='sd',na.rm=T)

colnames(fruit.stdev)[1]='Parent'

# Set parameters to make plot. This figure will have eight panels.

tiff(filename='Figure 2.tiff', width=8,height=4.5,units='in',res=600, compression='lzw')

par(mfrow=c(2,4),mar=c(5,5,1,1))

# A. Variation among individual seeds: mass as a function of area

plot(Mass~Area,data=fruits[fruits$Year==1983,],

xlab=expression(~Fruit~area~(cm^{2})),

xlim=c(10,40),ylim=c(0.5,3),

ylab='Fruit mass (g)',

pch=20, cex=0.7,cex.lab=0.9,

col=adjustcolor('black',alpha.f=0.2),

tcl=0.3, bty='l')

mtext('A',side=3,adj=0.07,line=-1.4, cex=0.8)

# Add lines for constant wing-loading

abline(a=0,b=0.04) # 40 mg/cm^2

abline(a=0,b=0.08) # 80 mg/cm^2

abline(a=0,b=0.16) # 160 mg/cm^2

text(x=c(39,39,22), # Add labels for constant wing-loading lines

y=c(1.45,2.9,3),

labels=c('40','80','160'),

offset=-1,

font=3)

# Calculate Pearson correlation coefficient

corval=round(cor(fruits[fruits$Year==1983,'Mass'],fruits[fruits$Year==1983,'Area'],

method='pearson',use='complete.obs'),3)

# Calculate significance of Pearson correlation

cor.test(fruits[fruits$Year==1983,'Mass'],fruits[fruits$Year==1983,'Area'],

alternative='t',method='pearson',exact=T)

# Plot Pearson correlation coefficient and significance

mtext(bquote('r'~"="~.(corval)~'***'),side=1,adj=0.85,line=-1.3,cex=0.7)

# B. Variation among individual seeds: rate of descent as a function of wingloading^0.5

plot(DescentRate~SqrtWL,data=fruits[fruits$Year==1983,],

xlab=expression(Wingloading^{0.5}~(mg^{0.5}/cm)),

ylab='Rate of descent (m/sec)',

xlim=c(5,13),ylim=c(1,3),

pch=20, cex=0.7,cex.lab=0.9,

col=adjustcolor('black',alpha.f=0.2),

tcl=0.3, bty='l')

mtext('B',side=3,adj=0.07,line=-1.4, cex=0.8)

# Calculate Pearson correlation coefficient

corval=round(cor(fruits[fruits$Year==1983,'DescentRate'],fruits[fruits$Year==1983,'SqrtWL'],

method='pearson',use='complete.obs'),3)

# Calculate significance of Pearson correlation

cor.test(fruits[fruits$Year==1983,'DescentRate'],fruits[fruits$Year==1983,'SqrtWL'],

alternative='t',method='pearson',exact=T)

# Plot Pearson correlation coefficient and significance

mtext(bquote('r'~"="~.(corval)~'***'),side=1,adj=0.85,line=-1.3,cex=0.7)

# C. Variation among individual seeds: actual dispersal distance from tower as a function of

# wingloading^0.5

plot(DistFrmTower~InvSqrtWL,data=fruits[fruits$Year==1983,],

xlab=expression(Wingloading^{-0.5}~(cm/mg^{0.5})),

ylab='Actual dispersal distance (m)',

xlim=c(0.07,0.17),ylim=c(5,50),

pch=20, cex=0.7,cex.lab=0.9,

col=adjustcolor('black',alpha.f=0.2),

tcl=0.3, bty='l')

mtext('C',side=3,adj=0.07,line=-1.4, cex=0.8)

#Calculate Pearson correlation coefficient

corval=round(cor(fruits[fruits$Year==1983,'DistFrmTower'],fruits[fruits$Year==1983,'InvSqrtWL'],

method='pearson',use='complete.obs'),3)

# Calculate significance of Pearson correlation

cor.test(fruits[fruits$Year==1983,'DistFrmTower'],fruits[fruits$Year==1983,'InvSqrtWL'],

alternative='t',method='pearson',exact=T)

# Plot Pearson correlation coefficient and significance

mtext(bquote('r'~"="~.(corval)~'**'),side=1,adj=0.85,line=-1.3,cex=0.7)

# D. Variation among individual seeds: actual dispersal distance from tower as a function of

# standard dispersal distance

plot(DistFrmTower~StdDist,data=fruits[fruits$Year==1983,],

xlab='Standard dispersal distance (m)',

ylab='Actual dispersal distance (m)',

xlim=c(12,38),ylim=c(5,50),

pch=20, cex=0.7,cex.lab=0.9,

col=adjustcolor('black',alpha.f=0.2),

tcl=0.3, bty='l')

mtext('D',side=3,adj=0.07,line=-1.4, cex=0.8)

# Calculate Pearson correlation coefficient

corval=round(cor(fruits[fruits$Year==1983,'DistFrmTower'],fruits[fruits$Year==1983,'StdDist'],

method='pearson',use='complete.obs'),3)

# Calculate significance of Pearson correlation

cor.test(fruits[fruits$Year==1983,'DistFrmTower'],fruits[fruits$Year==1983,'StdDist'],

alternative='t',method='pearson',exact=T)

# Plot Pearson correlation coefficient and significance

mtext(bquote('r'~"="~.(corval)~'***'),side=1,adj=0.85,line=-1.3,cex=0.7)

# E. Variation among parent trees: mass as a function of area

plot(Mass~Area,data=fruit.means,

xlab=expression(~Fruit~area~(cm^{2})),

ylab='Fruit mass (g)',

xlim=c(10,40),ylim=c(0.5,3),

pch=20,cex=0.7,cex.lab=0.9,

col=rainbow(20),

tcl=0.3, bty='l')

mtext('E',side=3,adj=0.07,line=-1.4, cex=0.8)

# Add lines for constant wing-loading

abline(a=0,b=0.04) # 40 mg/cm^2

abline(a=0,b=0.08) # 80 mg/cm^2

abline(a=0,b=0.16) # 160 mg/cm^2

text(x=c(39,39,22), # Add labels for constant wing-loading lines

y=c(1.45,2.9,3),

labels=c('40','80','160'),

offset=-1,

font=3)

# Plot vertical bars for standard deviation around the mean

arrows(x0=fruit.means$Area,y0=fruit.means$Mass-fruit.stdev$Mass,

x1=fruit.means$Area,y1=fruit.means$Mass+fruit.stdev$Mass,

length=0.02, angle=90,code=3,

col=rainbow(20))

# Plot horizontal bars for standard deviation around the mean

arrows(x0=fruit.means$Area-fruit.stdev$Area,y0=fruit.means$Mass,

x1=fruit.means$Area+fruit.stdev$Area,y1=fruit.means$Mass,

length=0.02, angle=90,code=3,

col=rainbow(20))

#Calculate Pearson correlation coefficient

corval=round(cor(fruit.means$Mass, fruit.means$Area,

method='pearson',use='complete.obs'),3)

# Calculate significance of Pearson correlation

cor.test(fruit.means$Mass, fruit.means$Area,

alternative='t',method='pearson',exact=T)

# Plot Pearson correlation coefficient and significance

mtext(bquote('r'~"="~.(corval)~'**'),side=1,adj=0.85,line=-1.3,cex=0.7)

# F. Variation among parent trees: rate of descent as a function of wingloading^0.5

plot(DescentRate~SqrtWL,data=fruit.means,

xlab=expression(Wingloading^{0.5}~(mg^{0.5}/cm)),

ylab='Rate of descent (m/sec)',

xlim=c(5,13),ylim=c(1,3),

pch=20,cex=0.7,cex.lab=0.9,

col=rainbow(20),

tcl=0.3, bty='l')

mtext('F',side=3,adj=0.07,line=-1.4, cex=0.8)

# Plot vertical bars for standard deviation around the mean

arrows(x0=fruit.means$SqrtWL,y0=fruit.means$DescentRate-fruit.stdev$DescentRate,

x1=fruit.means$SqrtWL,y1=fruit.means$DescentRate+fruit.stdev$DescentRate,

length=0.02, angle=90,code=3,

col=rainbow(20))

# Plot horizontal bars for standard deviation around the mean

arrows(x0=fruit.means$SqrtWL-fruit.stdev$SqrtWL,y0=fruit.means$DescentRate,

x1=fruit.means$SqrtWL+fruit.stdev$SqrtWL,y1=fruit.means$DescentRate,

length=0.02, angle=90,code=3,

col=rainbow(20))

#Calculate Pearson correlation coefficient

corval=round(cor(fruit.means$DescentRate, fruit.means$SqrtWL,

method='pearson',use='complete.obs'),3)

# Calculate significance of Pearson correlation

cor.test(fruit.means$DescentRate, fruit.means$SqrtWL,

alternative='t',method='pearson',exact=T)

# Plot Pearson correlation coefficient and significance

mtext(bquote('r'~"="~.(corval)~'***'),side=1,adj=0.85,line=-1.3,cex=0.7)

# G. Variation among parent trees: actual dispersal distance from tower as a function of

# wingloading^0.5

plot(DistFrmTower~InvSqrtWL,data=fruit.means,

xlab=expression(Wing~loading^{-0.5}~(mg/cm^{2})),

ylab='Actual dispersal distance (m)',

xlim=c(0.07,0.17),ylim=c(5,50),

pch=20,cex=0.7,cex.lab=0.9,

col=rainbow(20),

tcl=0.3, bty='l')

mtext('G',side=3,adj=0.07,line=-1.4, cex=0.8)

# Plot vertical bars for standard deviation around the mean

arrows(x0=fruit.means$InvSqrtWL,y0=fruit.means$DistFrmTower-fruit.stdev$DistFrmTower,

x1=fruit.means$InvSqrtWL,y1=fruit.means$DistFrmTower+fruit.stdev$DistFrmTower,

length=0.02, angle=90,code=3,

col=rainbow(20))

# Plot horizontal bars for standard deviation around the mean

arrows(x0=fruit.means$InvSqrtWL-fruit.stdev$InvSqrtWL,y0=fruit.means$DistFrmTower,

x1=fruit.means$InvSqrtWL+fruit.stdev$InvSqrtWL,y1=fruit.means$DistFrmTower,

length=0.02, angle=90,code=3,

col=rainbow(20))

#Calculate Pearson correlation coefficient

corval=round(cor(fruit.means$DistFrmTower, fruit.means$InvSqrtWL,

method='pearson',use='complete.obs'),3)

# Calculate significance of Pearson correlation

cor.test(fruit.means$DistFrmTower, fruit.means$InvSqrtWL,

alternative='t',method='pearson',exact=T)

# Plot Pearson correlation coefficient and significance

mtext(bquote('r'~"="~.(corval)~'*'),side=1,adj=0.85,line=-1.3,cex=0.7)

# H. Variation among parent trees: actual dispersal distance from tower as a function of

# standard dispersal distance

plot(DistFrmTower~StdDist,data=fruit.means,

xlab='Standard dispersal distance (m)',

ylab='Actual dispersal distance (m)',

xlim=c(12,38),ylim=c(5,50),

pch=20,cex=0.7,cex.lab=0.9,

col=rainbow(20),

tcl=0.3, bty='l')

mtext('H',side=3,adj=0.07,line=-1.4, cex=0.8)

# Plot vertical bars for standard deviation around the mean

arrows(x0=fruit.means$StdDist,y0=fruit.means$DistFrmTower-fruit.stdev$DistFrmTower,

x1=fruit.means$StdDist,y1=fruit.means$DistFrmTower+fruit.stdev$DistFrmTower,

length=0.02, angle=90,code=3,

col=rainbow(20))

# Plot horizontal bars for standard deviation around the mean

arrows(x0=fruit.means$StdDist-fruit.stdev$StdDist,y0=fruit.means$DistFrmTower,

x1=fruit.means$StdDist+fruit.stdev$StdDist,y1=fruit.means$DistFrmTower,

length=0.02, angle=90,code=3,

col=rainbow(20))

#Calculate Pearson correlation coefficient

corval=round(cor(fruit.means$DistFrmTower, fruit.means$StdDist,

method='pearson',use='complete.obs'),3)

# Calculate significance of Pearson correlation

cor.test(fruit.means$DistFrmTower, fruit.means$StdDist,

alternative='t',method='pearson',exact=T)

# Plot Pearson correlation coefficient and significance

mtext(bquote('r'~"="~.(corval)~'***'),side=1,adj=0.85,line=-1.3,cex=0.7)

dev.off()

#### Figure 3 ####

#### Interannual variation in fruit traits, showing the relationship between trait values in

#### 1980 Year 1 (x-axis) and 1983 Year 2 (y-axis).

# Load data

fruits <- read.csv('Individual fruits.csv')

# For each parent tree, calculate the mean fruit mass, area, and wingloading in 1980 (Year 1)

# and 1983 (Year 2)

fruit.means80=aggregate.data.frame(fruits[fruits$Year==1980,

c('Mass','Area','Wingloading')],

by=list(fruits[fruits$Year==1980,'Parent']),FUN='mean',na.rm=T)

colnames(fruit.means80)[1]='Parent'

fruit.means83=aggregate.data.frame(fruits[fruits$Year==1983,

c('Mass','Area','Wingloading')],

by=list(fruits[fruits$Year==1983,'Parent']),FUN='mean',na.rm=T)

colnames(fruit.means83)[1]='Parent'

# For each parent tree, calculate the standard deviation of fruit mass, area, and wingloading

# in 1980 (Year 1) and 1983 (Year 2)

fruit.stdev80=aggregate.data.frame(fruits[fruits$Year==1980,

c('Mass','Area','Wingloading')],

by=list(fruits[fruits$Year==1980,'Parent']),FUN='sd',na.rm=T)

colnames(fruit.stdev80)[1]='Parent'

fruit.stdev83=aggregate.data.frame(fruits[fruits$Year==1983,

c('Mass','Area','Wingloading')],

by=list(fruits[fruits$Year==1983,'Parent']),FUN='sd',na.rm=T)

colnames(fruit.stdev83)[1]='Parent'

# Set parameters to make plot. This figure will have three panels.

tiff(filename='Figure 3.tiff', width=8,height=2.25,units='in',res=600, compression='lzw')

par(mfrow=c(1,3),pty='s',mar=c(3,5,1,1),oma=c(1,0,0,0))

# A. Fruit mass in Year 1 vs mass in Year 2

plot(fruit.means80$Mass,fruit.means83$Mass[1:18],

xlab=NA,

ylab=NA,

xlim=c(1,3),ylim=c(1,3),

pch=20, asp=1, cex.axis=1,

col=rainbow(20)[1:18],

tcl=0.3, bty='l')

mtext('A. Mass (g)',side=3,adj=0.07,line=-1.4,cex=0.6)

mtext('Year 2',side=2,line=3)

# Plot a dashed 1-1 line

abline(a=0,b=1,lty=2)

# Plot vertical bars for standard deviation around the mean

arrows(x0=fruit.means80$Mass,y0=fruit.means83$Mass[1:18]-fruit.stdev83$Mass[1:18],

x1=fruit.means80$Mass,y1=fruit.means83$Mass[1:18]+fruit.stdev83$Mass[1:18],

length=0.02, angle=90,code=3,

col=rainbow(20)[1:18])

# Plot horizontal bars for standard deviation around the mean

arrows(x0=fruit.means80$Mass-fruit.stdev80$Mass,y0=fruit.means83$Mass[1:18],

x1=fruit.means80$Mass+fruit.stdev80$Mass,y1=fruit.means83$Mass[1:18],

length=0.02, angle=90,code=3,

col=rainbow(20)[1:18])

# Calculate Pearson correlation coefficient

corval=round(cor(fruit.means80$Mass,fruit.means83$Mass[1:18],

method='pearson', use='complete.obs'),3)

# Calculate significance of Pearson correlation

cor.test(fruit.means80$Mass,fruit.means83$Mass[1:18],alternative='t',

method='pearson',exact=T)

# Plot significance of Pearson correlation

mtext(bquote('r'~"="~.(format(corval,nsmall=3))~'***'),side=1,adj=0.85,line=-1.3, cex=0.7)

# B. Fruit area in Year 1 vs area in Year 2

plot(fruit.means80$Area,fruit.means83$Area[1:18],

xlab=NA,

ylab=NA,

xlim=c(10,40),ylim=c(10,40),

pch=20, asp=1,cex.axis=1,

col=rainbow(20)[1:18],

tcl=0.3, bty='l')

mtext(expression('B.'~Area~(cm^{2})),side=3,adj=0.07,line=-1.4,cex=0.6)

mtext('Year 1',side=1,line=3)

# Plot a dashed 1-1 line

abline(a=0,b=1,lty=2)

# Plot vertical bars for standard deviation around the mean

arrows(x0=fruit.means80$Area,

y0=fruit.means83$Area[1:18]-fruit.stdev83$Area[1:18],

x1=fruit.means80$Area,

y1=fruit.means83$Area[1:18]+fruit.stdev83$Area[1:18],

length=0.02, angle=90,code=3,

col=rainbow(20)[1:18])

# Plot horizontal bars for standard deviation around the mean

arrows(x0=fruit.means80$Area-fruit.stdev80$Area,

y0=fruit.means83$Area[1:18],

x1=fruit.means80$Area+fruit.stdev80$Area,

y1=fruit.means83$Area[1:18],

length=0.02, angle=90,code=3,

col=rainbow(20)[1:18])

# Calculate Pearson correlation coefficient

corval=round(cor(fruit.means80$Area,fruit.means83$Area[1:18],

method='pearson', use='complete.obs'),3)

# Calculate significance of Pearson correlation

cor.test(fruit.means80$Area,fruit.means83$Area[1:18],

alternative='t',method='pearson',exact=T)

# Plot significance of Pearson correlation

mtext(bquote('r'~"="~.(corval)~'***'),side=1,adj=0.85,line=-1.3, cex=0.7)

# C. Fruit wingloading in Year 1 vs wingloading in Year 2

plot(fruit.means80$Wingloading,fruit.means83$Wingloading[1:18],

xlab=NA,

ylab=NA,

xlim=c(40,140),ylim=c(40,140),

pch=20, asp=1,cex.axis=1,

col=rainbow(20)[1:18],

tcl=0.3, bty='l')

mtext(expression('C.'~Wing-loading~(mg/cm^{2})),side=3,adj=0.4,line=-1.4,cex=0.6)

# Plot a dashed 1-1 line

abline(a=0,b=1,lty=2)

# Plot vertical bars for standard deviation around the mean

arrows(x0=fruit.means80$Wingloading,

y0=fruit.means83$Wingloading[1:18]-fruit.stdev83$Wingloading[1:18],

x1=fruit.means80$Wingloading,

y1=fruit.means83$Wingloading[1:18]+fruit.stdev83$Wingloading[1:18],

length=0.02, angle=90,code=3,

col=rainbow(20)[1:18])

# Plot horizontal bars for standard deviation around the mean

arrows(x0=fruit.means80$Wingloading-fruit.stdev80$Wingloading,

y0=fruit.means83$Wingloading[1:18],

x1=fruit.means80$Wingloading+fruit.stdev80$Wingloading,

y1=fruit.means83$Wingloading[1:18],

length=0.02, angle=90,code=3,

col=rainbow(20)[1:18])

# Calculate Pearson correlation coefficient

corval=round(cor(fruit.means80$Wingloading,fruit.means83$Wingloading[1:18],

method='pearson', use='complete.obs'),3)

# Calculate significance of Pearson correlation

cor.test(fruit.means80$Wingloading,fruit.means83$Wingloading[1:18],

alternative='t',method='pearson',exact=T)

# Plot significance of Pearson correlation

mtext(bquote('r'~"="~.(corval)~'**'),side=1,adj=0.85,line=-1.3, cex=0.7)

dev.off()

#### Figure 6 ####

#### Variation in seed dispersal distance distributions among six parent trees for

#### seeds released from a tower (A), seeds naturally dispersed in the southernmost

#### sector of the seed shadows of parent trees (B), and seeds naturally dispersed in the

#### entire seed shadows of parent trees (C).

# Load data

fruits <- read.csv('Individual fruits.csv')

treetraits <- read.csv('Parent tree information.csv')

seeddist <- read.csv('Measured dispersal distances.csv')

# Add half trunk diameter to measured distance from parent trunk

for (i in 2:7){

seeddist[seeddist$Parent==i,'Dist']<- (seeddist[seeddist$Parent==i,'Dist']

+0.5*treetraits[treetraits$Parent==i,'DBH']/100)

}

# Correct for decreased sampling effort with increasing distance from tree

# Define an "effort factor": the area actually sampled at that distance divided

# by the total area at that distance

seeddist$eff.fac <- seeddist$Area/(seeddist$Dist^2-(seeddist$Dist-1)^2)

# Divide measurement by the effort factor

seeddist$seeds.corrected <- seeddist$Seeds/seeddist$eff.fac

# For each parent, calculate the cumulative percent of seeds dispersed with distance from the

# tower, from the tree in all directions, and from the tree only including the

# southern direction.

# Make a data frame to store results (six parent trees, 100 m per tree)

seed.shadows=matrix(ncol=5,nrow=600,data=NA)

seed.shadows=data.frame(seed.shadows)

colnames(seed.shadows)= c('Parent','Distance','Tower','Tree.all','Tree.south')

seed.shadows$Distance = rep(c(1:100),6)

# Using a loop, calculated cumulative dispersal for each tree:

for (i in 2:7){

toweri=fruits[fruits$Parent==i,]

treei=seeddist[seeddist$Parent==i,]

# For each distance, calculate cumulative proportion of seeds at that distance

for (j in 1:100){

# Record current parent tree number

seed.shadows$Parent[(i-2)*100 + j] = i

# Calculate proportion of seeds at current distance from tower

seed.shadows$Tower[(i-2)*100 + j] = (100*length(toweri[ !is.na(toweri$DistFrmTower)

& toweri$DistFrmTower <= j,'DistFrmTower'])

/ length(toweri[ !is.na(toweri$DistFrmTower),'DistFrmTower']))

# Calculate proportion of seeds at current distance from tree (all directions)

seed.shadows$Tree.all[(i-2)*100 + j] = (100*sum(treei[treei$Dist <= j, 'seeds.corrected'])

/ sum(treei$seeds.corrected))

# Calculate proportion of seeds at current distance from tree (South only)

seed.shadows$Tree.south[(i-2)*100 + j] = (100*sum(treei[treei$Dir == 180 &

treei$Dist <= j, 'seeds.corrected'])

/ sum(treei[treei$Dir == 180, 'seeds.corrected']))

}

}

# For each parent tree, calculate maximum seed dispersal distance from the tower, from the tree

# in all directions, and from the tree in the southern directions only

# Make a data frame to store results (six parent trees, 100 m per tree)

maxdist = matrix(ncol=4,nrow=6,data=NA)

maxdist = data.frame(maxdist)

colnames(maxdist) = c('Parent','Tower','Tree.all','Tree.south')

# Run a loop to calculate maximum distance for each parent

for (i in 2:7) {

# Record correct parent number

maxdist$Parent[i-1] = i

# Subset current parent from tower and tree data

toweri=fruits[fruits$Parent==i,]

treei=seeddist[seeddist$Parent==i & seeddist$Seeds > 0,]

#Calculate maximum distances

maxdist$Tower[i-1] = max(toweri$DistFrmTower,na.rm=T)

maxdist$Tree.all[i-1] = max(treei$Dist,na.rm=T)

maxdist$Tree.south[i-1] = max(treei[treei$Dir == 180,'Dist'],na.rm=T)

}

# Set parameters to make plot. This figure will have three panels.

tiff(filename='Figure 5.tiff', width=4,height=6.75,units='in',res=600, compression='lzw')

par(mfrow=c(3,1),mar=c(2,1,1,1), oma=c(3,3,0,0))

# Choose six line types and colors so that it is easy to distinguish between the parent trees.

ltys=c(1,1,1,2,2,2)

parent.col=colors()[c(24, 33, 150, 258, 26, 640)]

# A. Cumulative seed dispersal: dispersal from tower

plot(Tower~Distance, data=seed.shadows,

type = 'n', # First, create a blank plot of appropriate size and label axes

xlim = c(1,100),

ylim = c(0,100),

xlab = NA,

ylab = NA,

xaxt ='n',yaxt='n',bty='n',

log = 'x')

axis(side=1,at=c(1,10,25,50,100), pos=0, tcl=0.3)

axis(side=2,at=seq(from=0, to=100, by=20), pos=1, tcl=0.3)

mtext('A', side=3, line=-2, adj=0.1)

# Use a loop to plot data for each parent tree in a separate color

for (i in 2:7){

# Subset one parent tree from seed shadow data

toweri=fruits[fruits$Parent==i,]

treei=seed.shadows[seed.shadows$Parent == i,]

# Plot a line for each parent tree

lines(Tower~Distance,data=treei[treei$Tower < 100,],

col=parent.col[i-1], lty = ltys[i-1],lwd=1.2)

# Plot a point at the maximum distance for each parent tree

points(maxdist$Tower[i-1],100,pch=20,col=parent.col[i-1])

# Plot a vertical line for the mean dispersal distance for each parent tree

abline(v=mean(toweri$DistFrmTower,na.rm=T),

col=parent.col[i-1], lty = ltys[i-1],lwd=1.2)

}

# Create a legend

legend(x='bottomleft',y=NULL,bty='n',

legend = c(2:7), title= 'Parent tree',

inset = 0.1,

lty = ltys,

lwd = 1.2,

col = parent.col)

# B. Cumulative seed dispersal: dispersal from parent trees (Southern sector)

plot(Tower~Distance, data=seed.shadows,

type = 'n', # First, create a blank plot of appropriate size and label axes

xlim = c(1,100),

ylim = c(0,100),

xlab = NA,

ylab = NA,

xaxt='n',yaxt='n',bty='n', log='x')

mtext('B', side=3, line=-2, adj=0.1)

axis(side=1,at=c(1,10,25,50,100), pos=0, tcl=0.3)

axis(side=2,at=seq(from=0, to=100, by=20), pos=1, tcl=0.3)

# Use a loop to plot data for each parent tree in a separate color

for (i in 2:7){

# Subset one parent tree from seed shadow data

tree.disti=seeddist[seeddist$Parent==i,]

treei=seed.shadows[seed.shadows$Parent == i,]

# Plot a line for each parent tree

lines(Tree.south~Distance,data=treei[treei$Tree.south < 100,],

col=parent.col[i-1],lty=ltys[i-1],lwd=1.2)

# Plot a point at the maximum distance for each parent tree

points(maxdist$Tree.south[i-1],100,pch=20,col=parent.col[i-1])

# Plot a vertical line for the mean dispersal distance for each parent tree

abline(v=weighted.mean(x=tree.disti[tree.disti$Dir==180,'Dist'],

w=tree.disti[tree.disti$Dir==180,'seeds.corrected']),

col=parent.col[i-1],lty=ltys[i-1],lwd=1.2)

}

# C. Cumulative seed dispersal: dispersal from parent trees (all sectors)

plot(Tower~Distance, data=seed.shadows,

type = 'n', # First, create a blank plot of appropriate size and label axes

xlim = c(1,100),

ylim = c(0,100),

xlab = NA,

ylab = NA,

xaxt='n',yaxt='n',bty='n', log = 'x')

mtext('C', side=3, line=-2, adj=0.1)

axis(side=1,at=c(1,10,25,50,100), pos=0, tcl=0.3)

axis(side=2,at=seq(from=0, to=100, by=20), pos=1, tcl=0.3)

# Use a loop to plot data for each parent tree in a separate color

for (i in 2:7){

# Subset one parent tree from seed shadow data

tree.disti=seeddist[seeddist$Parent == i,]

treei=seed.shadows[seed.shadows$Parent == i,]

# Plot a line for each parent tree

lines(Tree.all~Distance,data=treei[treei$Tree.all < 100,],

col=parent.col[i-1],lty=ltys[i-1],lwd=1.2)

# Plot a point at the maximum distance for each parent tree

points(maxdist$Tree.all[i-1],100,pch=20,col=parent.col[i-1])

# Plot a vertical line for the mean dispersal distance for each parent tree

abline(v=weighted.mean(x=tree.disti[,'Dist'],

w=tree.disti[,'seeds.corrected']),

col=parent.col[i-1],lty=ltys[i-1],lwd=1.2)

}

# Label axes

mtext('Dispersal distance (m)', side=1, outer=T, line=1.5)

mtext('Cumulative percent of seeds dispersed', side=2, outer=T, line=1.5)

dev.off()

#### Figure 7 ####

#### For each parent tree, percent of total number of offspring as a function of distance from

#### the parent tree for different life stages.

# Load data

seedlings=read.csv('Seed seedling and sapling distributions.csv')

# When no seedlings were recorded, replace NA with 0

seedlings[is.na(seedlings)]=0

# Set parameters to make plot. This figure will have six panels.

tiff(filename='Figure 6.tiff', width=8,height=6.75,units='in',res=600, compression='lzw')

par(mfrow=c(3,2),mar=c(3,2,1,1), oma=c(2,2,1,1))

# Plot first panel, with legend

# Subset only first parent tree

parenti=seedlings[seedlings$Parent==2,]

# Calculate percent of seeds at each distance

pct.seed=100*parenti$Seeds/sum(parenti$Seeds, na.rm=T)

# Calculate percent of seedlings at each distance

pct.seedling=100*parenti$Seedlings/sum(parenti$Seedlings, na.rm=T)

# Calculate percent of saplings at each distance

pct.sapling=100*parenti$Saplings/sum(parenti$Saplings, na.rm=T)

# Make plot

plot(pct.seed~parenti$Dist,

type='o',

xlab=NA,

ylab=NA,

pch=19,

ylim=c(0,50),

xlim = c(0,120),

xaxt='n',yaxt='n',bty='n')

points(pct.seedling~parenti$Dist,

type='o',pch=1,col = 'red')

points(pct.sapling~parenti$Dist,

type='o', pch=18,col = 'blue')

mtext('A. Parent 2',side=3,adj=0.07,line=-1.4)

axis(side=1,at=seq(from=0, to=120, by=20), pos=0, tcl=0.3, cex.axis=1.4)

axis(side=2,at=seq(from=0, to=50, by=25), pos=0, tcl=0.3, cex.axis=1.4)

legend(x='topright',y=NULL,c('Seeds','Seedlings','Saplings'),bty='n',pch=c(19,1,18),

col=c('black','red','blue'), cex=1.4)

# Plot other panels using a loop

for (i in 3:7) {

# Subset one parent tree

parenti=seedlings[seedlings$Parent==i,]

# Calculate percent of seeds at each distance

pct.seed=100*parenti$Seeds/sum(parenti$Seeds, na.rm=T)

# Calculate percent of seedlings at each distance

pct.seedling=100*parenti$Seedlings/sum(parenti$Seedlings, na.rm=T)

# Calculate percent of saplings at each distance

pct.sapling=100*parenti$Saplings/sum(parenti$Saplings, na.rm=T)

# Make plot

plot(pct.seed~parenti$Dist,

type='o',

xlab=NA,

ylab=NA,

pch=19,

ylim=c(0,50),

xlim = c(0,120),

xaxt='n',yaxt='n',bty='n')

points(pct.seedling~parenti$Dist,

type='o',pch=1,col = 'red')

points(pct.sapling~parenti$Dist,

type='o', pch=18,col = 'blue')

mtext(c('B. Parent 3','C. Parent 4','D. Parent 5','E. Parent 6','F. Parent 7')[i-2],

side=3,adj=0.07,line=-1.4)

axis(side=1,at=seq(from=0, to=120, by=20), pos=0, tcl=0.3, cex.axis=1.4)

axis(side=2,at=seq(from=0, to=50, by=25), pos=0, tcl=0.3, cex.axis=1.4)

}

# Label axes

mtext('Distance (m)', side=1, outer=T)

mtext('Percentage', side=2, outer=T)

dev.off()

#### Figure 8 ####

#### Overall distributions of seeds, seedlings, and saplings as a function of distance from

#### edge of parent trunk.

# Load data

seedlings <- read.csv('Seed seedling and sapling distributions.csv')

treetraits <- read.csv('Parent tree information.csv')

# When no seedlings were recorded, replace NA with 0

seedlings[is.na(seedlings)]=0

# Add half trunk diameter to measured distance from parent trunk

for (i in 2:7){

seedlings[seedlings$Parent==i,'Dist'] <- seedlings[seedlings$Parent==i,'Dist'] +

0.5*treetraits[treetraits$Parent==i,'DBH']/100

}

# Calculate the total numbers and percents of seeds, seedlings, and saplings at distance

# intervals of 0.25 m from 0 m to 37.5 m, then 37.5-45 m, 45-55 m, 55-70 m, and 70-90 m:

# Create data frames to store results

seeds.tot=rep(NA,length(c(seq(2.5,37.5,5),45,55,70,90))) # Total number of seeds

seedlings.tot=rep(NA,length(c(seq(2.5,37.5,5),45,55,70,90))) # Total number of seedlings

saplings.tot=rep(NA,length(c(seq(2.5,37.5,5),45,55,70,90))) # Total number of saplings

seed.pct=rep(NA,length(c(seq(2.5,37.5,5),45,55,70,90))) # Percent of seeds

seedling.pct=rep(NA,length(c(seq(2.5,37.5,5),45,55,70,90))) # Percent of seedlings

sapling.pct=rep(NA,length(c(seq(2.5,37.5,5),45,55,70,90))) # Percent of saplings

seed.pctile=rep(NA,length(c(seq(2.5,37.5,5),45,55,70,90))) # Percentile of seeds

# Use a loop to calculate numers, percents, and percentiles

for(i in 1:length(c(seq(2.5,37.5,5),45,55,70,90))) {

num=c(seq(2.5,37.5,5),45,55,70,90)[i] # Upper distance bound for current iteration

prev = c(0,seq(2.5,37.5,5),45,55,70)[i] # Lower distance bound for current iteration

# Calculate total number of seeds, percent of seeds, and percentile of seeds

seeds.tot[i]=sum(seedlings[seedlings$Dist <= num

& seedlings$Dist > prev ,'Seeds'],na.rm=T)

seed.pct[i]=100*seeds.tot[i]/sum(seedlings$'Seeds')/(num-prev)

seed.pctile[i]= 100*sum(seeds.tot[1:i])/sum(seedlings$'Seeds',na.rm=T)

# Calculate total number of seedlings and percent of seedlings

seedlings.tot[i]=sum(seedlings[seedlings$Dist <= num

& seedlings$Dist > prev,'Seedlings'],na.rm=T)

seedling.pct[i]=100*seedlings.tot[i]/sum(seedlings$'Seedlings',na.rm=T)/(num-prev)

# Calculate total number of saplings and percent of seedlings

saplings.tot[i]=sum(seedlings[seedlings$Dist <= num

& seedlings$Dist > prev,'Saplings'],na.rm=T)

sapling.pct[i]=100*saplings.tot[i]/sum(seedlings$'Saplings',na.rm=T)/(num-prev)

}

# Set parameters to make plot. This figure will have three panels.

tiff(filename='Figure 7.tiff', width=4,height=6.75,units='in',res=600, compression='lzw')

par(mfrow=c(3,1),mar=c(4,4,1,1),oma=c(0,1,0,0))

parent.col=colors()[c(24, 33, 150, 258, 26, 640)]

# Panel A. Proportions of seeds, seedlings, and saplings as a function of distance from the

# parent tree for all trees combined.

plot(seed.pct~c(seq(2.5,37.5,5),45,55,70,90),

type='b',

xlab='Distance (m)',

ylab='Percentage (%/m)',

pch=19,

ylim=c(0,5),

xlim=c(0,100),

xaxt='n',yaxt='n', bty='n',

col=parent.col[1],

cex.lab=1.4)

points(seedling.pct~c(seq(2.5,37.5,5),45,55,70,90), type='b',pch=1,col=parent.col[2])

points(sapling.pct~c(seq(2.5,37.5,5),45,55,70,90), type='b', pch=18,col=parent.col[5])

mtext('A',side=3,adj=0.07,line=-2)

axis(side=1,at=seq(from=0, to=100, by=20), pos=0, tcl=0.3, cex.axis=1.4)

axis(side=2,at=seq(from=0, to=5, by=1), pos=0, tcl=0.3, cex.axis=1.4)

# Make legend

legend(x='topright',y=NULL,c('Seeds','Seedlings','Saplings'),bty='n',pch=c(19,1,18),

col=parent.col[c(1,2,5)], cex=1.4)

# Panel B. Seedlings per seed and saplings per seed as a function of distance.

plot((seedlings.tot/seeds.tot)~c(seq(2.5,37.5,5),45,55,70,90),

type='b',

pch=1,

xlab='Distance (m)',

ylab='Individuals per seed',

ylim=c(0,0.03), xlim=c(0,100),

xaxt='n',yaxt='n', bty='n', col=parent.col[2],

cex.lab=1.4)

points((saplings.tot/seeds.tot)~c(seq(2.5,37.5,5),45,55,70,90),type='b',

pch=18, col=parent.col[5])

mtext('B',side=3,adj=0.07,line=-2)

axis(side=1,at=seq(from=0, to=100, by=20), pos=0, tcl=0.3, cex.axis=1.4)

axis(side=2,at=seq(from=0, to=0.03, by=0.01), pos=0, tcl=0.3, cex.axis=1.4)

# Panel C. Seedlings per seed and saplings per seed as a function of the dispersal

# distance percentile.

plot((seedlings.tot/seeds.tot)~seed.pctile,

type='b',

pch=1,

xlab='Seed dispersal distance percentile (%)',

ylab='Individuals per seed',

ylim=c(0,0.03), xlim=c(0,100),

xaxt='n',yaxt='n', bty='n',col=parent.col[2],

cex.lab=1.4)

points((saplings.tot/seeds.tot)~seed.pctile,type='b',pch=18, col=parent.col[5])

mtext('C',side=3,adj=0.07,line=-2)

axis(side=1,at=seq(from=0, to=100, by=20), pos=0, tcl=0.3, cex.axis=1.4)

axis(side=2,at=seq(from=0, to=0.03, by=0.01), pos=0, tcl=0.3, cex.axis=1.4)

dev.off()

#### Figure S1 ####

#### Comparison among parent trees (one line per tree) of seed dispersal distance distributions

#### from the tower among parent trees.

# Load data

fruits <- read.csv('Individual fruits.csv')

# Set parameters to make plot. This figure will have one panel.

tiff(filename='Figure S1.tiff', width=4,height=4,units='in',res=600, compression='lzw')

par(mfrow=c(1,1))

# Create an empty plot of appropriate size for seed dispersal distance distribution.

plot(0,0,type='n',

xlim=c(0,55), ylim=c(0,1),

xlab='Distance from tower (m)',

ylab='Probability of dispersal',

tcl=0.3, bty='l')

# Use a loop to plot a CDF for each of 20 parents in a different color

for (i in 1:20) {

# Subset one parent at a time using Year 1 (1983) Data with measured distance from tower

parenti=fruits[fruits$Year==1983 & fruits$Parent==i & !(is.na(fruits$DistFrmTower)),]

# Use "density" function to create a smoothed, fitted probability distribution function

densfn = density(parenti$DistFrmTower)

# Calculate a cumulative distribution function using the probability density function

cumd = c() # Create a vector to store CDF values

# Use a loop to sum the values of the PDF up to each distance

for(j in 1:length(densfn$x)){

cumd[j]=sum(densfn$y[1:j])

}

# Normalize the PDF

cumd=cumd/max(cumd)

# Plot the CDF

lines(densfn$x,cumd,col=rainbow(20)[i])

points(max(densfn$x),1,col=rainbow(20)[i],pch=20)

}

dev.off()

#### Figure S2 ####

#### Seed shadows predicted from a mechanistic model of seed dispersal incorporating measured

#### intraspecific variation in seed and tree traits.

# Load data

load('Mechanistic dispersal results.RData')

# Set parameters to make plot. This figure will have six panels.

tiff(filename='Figure S2.tiff', width=9,height=7,units='in',res=600, compression='lzw')

par(mfrow=c(2,3),mar=c(2,2,1,1), oma=c(3,3,0,0))

for( i in 1:6){

plot(seed.x~seed.y, data=modelD.results[[i]],

col=adjustcolor('black',alpha.f=0.15),

xlab=NA,ylab=NA,

pch=20,

xlim=c(-150,150),ylim=c(-300,50),

tcl=0.3, bty='l',

cex.axis=1.2)

mtext(paste('Parent ',i+1),side=3,adj=0.07,line=-2)

points(c(0,0),pch=20,col='red',cex=2)

}

# Label axes

mtext('Seed position (m)', side=1, outer=T, line=1)

mtext('Seed position (m)', side=2, outer=T, line=1)

dev.off()

**R script 2 to fit models for variation in rate of descent and dispersal distances from the tower**

###### Intraspecific variation in seed dispersal of a Neotropical tree and its relationship to

###### fruit and tree traits.

###### Augspurger et al.

###### Variation in descent and dispersal.R ######

#### This file contains the code to fit alternative models to explain variation

#### among fruits in the rate of descent in still air and dispersal distances from the tower.

## Define a function to calculate R-squared for models with explanatory variables

## raised to a fixed power. Here, 'nvars' gives the number of variables, 'data' is the observed

## values, and 'model' is the predicted values. 'R2' is R-squared value and 'R2.a' is the

## adjusted R-squared value.

Fixed.Rsquared <- function(data,model,nvars){

SStot = sum((data - mean(data))^2)

SSres = sum((data-model)^2)

R2 = 1 - SSres/SStot

n=length(data)

R2.a = 1-(1-R2)*((n-1)/(n-nvars-1))

to.return = list(R2,R2.a)

return(to.return)

}

## 1. Load file of individual fruit data, and calculate summary statistics for each parent tree:

fruits <- read.csv('Individual fruits.csv')

# Ensure all fruits have complete measurements for descent rate, mass, area, and wingloading

fruits.narm=fruits[!(is.na(fruits$DescentRate)) & !(is.na(fruits$Mass))

& !(is.na(fruits$Area)) & !(is.na(fruits$Wingloading)), ]

# Also select only fruits that have complete measurements for dispersal distance

dist.narm=fruits[!(is.na(fruits$DescentRate)) & !(is.na(fruits$Mass))

& !(is.na(fruits$Area)) & !(is.na(fruits$Wingloading))

& !(is.na(fruits$DistFrmTower)), ]

# Calculate summary statistics for each parent tree:

# Make parent tree a factor

fruits.narm$Parent = as.factor(fruits.narm$Parent)

# Create data frame to store results for mean, median, and percentiles for each tree

mass.summary = data.frame(Parent=levels(fruits.narm$Parent), #for mass

mean = NA, q50 = NA, q75 = NA, q90 = NA, q95 = NA, q100 = NA)

area.summary = data.frame(Parent=levels(fruits.narm$Parent), #for area

mean = NA, q50 = NA, q75 = NA, q90 = NA, q95 = NA, q100 = NA)

wl.summary = data.frame(Parent=levels(fruits.narm$Parent), #for wingloading

mean = NA, q50 = NA, q75 = NA, q90 = NA, q95 = NA, q100 = NA)

dr.summary = data.frame(Parent=levels(fruits.narm$Parent), #for descent rate

mean = NA, q50 = NA, q75 = NA, q90 = NA, q95 = NA, q100 = NA)

dist.summary = data.frame(Parent=levels(fruits.narm$Parent), #for dispersal distance

mean = NA, q50 = NA, q75 = NA, q90 = NA, q95 = NA, q100 = NA)

# Run a loop to calculate values for each tree

for (i in 1:length(levels(fruits.narm$Parent))) {

# choose one tree at a time

treei=fruits.narm[fruits.narm$Parent == levels(fruits.narm$Parent)[i],]

# calculate mass mean and percentiles

mass.summary$mean[i] = mean(log(treei$Mass))

mass.summary[i,c('q50','q75','q90','q95','q100')] = quantile(log(treei$Mass),

probs = c(0.50, 0.75, 0.90, 0.95, 1.0))

# calculate area mean and percentiles

area.summary$mean[i] = mean(log(treei$Area))

area.summary[i,c('q50','q75','q90','q95','q100')] = quantile(log(treei$Area),

probs = c(0.50, 0.75, 0.90, 0.95, 1.0))

# calculate wing-loading mean and percentiles

wl.summary$mean[i] = mean(log(treei$Wingloading))

wl.summary[i,c('q50','q75','q90','q95','q100')] = quantile(log(treei$Wingloading),

probs = c(0.50, 0.75, 0.90, 0.95, 1.0))

# calculate descent rate mean and percentiles

dr.summary$mean[i] = mean(log(treei$DescentRate))

dr.summary[i,c('q50','q75','q90','q95','q100')] = quantile(log(treei$DescentRate),

probs = c(0.50, 0.25, 0.10, 0.05, 0.0))

# calculate dispersal distance mean and percentiles

dist.summary$mean[i] = mean(log(treei$DistFrmTower),na.rm=T)

dist.summary[i,c('q50','q75','q90','q95','q100')] = quantile(log(treei$DistFrmTower),

probs = c(0.50, 0.75, 0.90, 0.95, 1.0),na.rm=T)

}

## 2. Compare models to predict rate of descent for indivdual seeds.

descent.1=lm(log(DescentRate)~(1),

data=fruits.narm)

descent.2=lm(log(DescentRate)~offset(0.5*log(Wingloading)),

data=fruits.narm)

descent.3=lm(log(DescentRate)~log(Wingloading),

data=fruits.narm)

descent.4=lm(log(DescentRate)~log(Area),

data=fruits.narm)

descent.5=lm(log(DescentRate)~log(Mass),

data=fruits.narm)

descent.6=lm(log(DescentRate)~log(Mass)+log(Area),

data=fruits.narm)

# Compare the AIC values for these models:

descentAIC=AIC(descent.1,descent.2,descent.3,descent.4,descent.5,descent.6)

# Order by AIC value (smallest to largest)

descentAIC=descentAIC[order(descentAIC$AIC),]

descentAIC$dAIC <- descentAIC$AIC-descentAIC$AIC[1]

# descent.6 is the best model. View each model:

summary(descent.6)

summary(descent.3)

summary(descent.2)

Fixed.Rsquared(log(fruits.narm$DescentRate),fitted(descent.2),1)

summary(descent.5)

summary(descent.4)

summary(descent.1)

## 3. Compare models to predict mean, median, and qualtiles of rate of descent for each tree.

## Use: 1. Null model

## 2. The model predicted by first principles

## 3. The best model predicted from individual seed data

## Models for mean descent rate

# dr.mean1: First principles prediction: proportional to the square-root of wingloading.

# dr.mean2: The best model predicted from individual seed data

dr.mean1=descent.2$coefficients[1]+0.5*wl.summary$mean

dr.mean2=descent.6$coefficients[1]+descent.6$coefficients[2]*mass.summary$mean+descent.6$coefficients[3]*area.summary$mean

# Calculate Pearson correlation coefficient and significance

cortest1 <- cor.test(dr.summary$mean, dr.mean1,method='pearson',exact=T)

cortest1$estimate; cortest1$p.value

cortest2 <- cor.test(dr.summary$mean, dr.mean2,method='pearson',exact=T)

cortest2$estimate; cortest2$p.value

## Models for median descent rate

# dr.50q1: First principles prediction: proportional to the square-root of wingloading.

# dr.50q2: The best model predicted from individual seed data

dr.50q1=descent.2$coefficients[1]+0.5*wl.summary$q50

dr.50q2=descent.6$coefficients[1]+descent.6$coefficients[2]*mass.summary$q50+descent.6$coefficients[3]*area.summary$q50

# Calculate Pearson correlation coefficient and significance

cortest1 <- cor.test(dr.summary$q50, dr.50q1,method='pearson',exact=T)

cortest1$estimate; cortest1$p.value

cortest2 <- cor.test(dr.summary$q50, dr.50q2,method='pearson',exact=T)

cortest2$estimate; cortest2$p.value

## Models for the 75th percentile of descent rate

# dr.75q1: First principles prediction: proportional to the square-root of wingloading.

# dr.75q2: The best model predicted from individual seed data

dr.75q1=descent.2$coefficients[1]+0.5*wl.summary$q75

dr.75q2=descent.6$coefficients[1]+descent.6$coefficients[2]*mass.summary$q75+descent.6$coefficients[3]*area.summary$q75

# Calculate Pearson correlation coefficient and significance

cortest1 <- cor.test(dr.summary$q75, dr.75q1,method='pearson',exact=T)

cortest1$estimate; cortest1$p.value

cortest2 <- cor.test(dr.summary$q75, dr.75q2,method='pearson',exact=T)

cortest2$estimate; cortest2$p.value

## Models for the 90th percentile of descent rate

# dr.90q1: First principles prediction: proportional to the square-root of wingloading.

# dr.90q2: The best model predicted from individual seed data

dr.90q1=descent.2$coefficients[1]+0.5*wl.summary$q90

dr.90q2=descent.6$coefficients[1]+descent.6$coefficients[2]*mass.summary$q90+descent.6$coefficients[3]*area.summary$q90

# Calculate Pearson correlation coefficient and significance

cortest1 <- cor.test(dr.summary$q90, dr.90q1,method='pearson',exact=T)

cortest1$estimate; cortest1$p.value

cortest2 <- cor.test(dr.summary$q90, dr.90q2,method='pearson',exact=T)

cortest2$estimate; cortest2$p.value

## Models for the 95th percentile of descent rate

# dr.95q1: First principles prediction: proportional to the square-root of wingloading.

# dr.95q2: The best model predicted from individual seed data

dr.95q1=descent.2$coefficients[1]+0.5*wl.summary$q95

dr.95q2=descent.6$coefficients[1]+descent.6$coefficients[2]*mass.summary$q95+descent.6$coefficients[3]*area.summary$q95

# Calculate Pearson correlation coefficient and significance

cortest1 <- cor.test(dr.summary$q95, dr.95q1,method='pearson',exact=T)

cortest1$estimate; cortest1$p.value

cortest2 <- cor.test(dr.summary$q95, dr.95q2,method='pearson',exact=T)

cortest2$estimate; cortest2$p.value

## Models for maximum descent rate

# dr.100q1: First principles prediction: proportional to the square-root of wingloading.

# dr.100q2: The best model predicted from individual seed data

dr.100q1=descent.2$coefficients[1]+0.5*wl.summary$q100

dr.100q2=descent.6$coefficients[1]+descent.6$coefficients[2]*mass.summary$q100+descent.6$coefficients[3]*area.summary$q100

# Calculate Pearson correlation coefficient and significance

cortest1 <- cor.test(dr.summary$q100, dr.100q1,method='pearson',exact=T)

cortest1$estimate; cortest1$p.value

cortest2 <- cor.test(dr.summary$q100, dr.100q2,method='pearson',exact=T)

cortest2$estimate; cortest2$p.value

## 4. Compare models to predict dispersal distance for indivdual seeds.

## Parent tree is included as a random effect for all models.

# dist.1: Null model.

# dist.2: Proportional to the square-root of wingloading.

# dist.3: Power function of wingloading.

# dist.4: Power function of area.

# dist.5: Power function of mass.

# dist.6: Power function of mass and area.

# dist.7: Inversely proportional to descent rate.

# dist.8: Power function of descent rate.

# dist.9: Power function of wingloading and descent rate.

# dist.10: Power function of area and descent rate.

# dist.11: Power function of mass and descent rate.

# dist.12: Power function of mass, area, and descent rate.

dist.1=lm(log(DistFrmTower)~1,

data=dist.narm)

dist.2=lm(log(DistFrmTower)~offset(-0.5*log(Wingloading)),

data=dist.narm)

dist.3=lm(log(DistFrmTower)~log(Wingloading),

data=dist.narm)

dist.4=lm(log(DistFrmTower)~log(Area),

data=dist.narm)

dist.5=lm(log(DistFrmTower)~log(Mass),

data=dist.narm)

dist.6=lm(log(DistFrmTower)~log(Mass)+log(Area),

data=dist.narm)

dist.7=lm(log(DistFrmTower)~offset(-1*log(DescentRate)),

data=dist.narm)

dist.8=lm(log(DistFrmTower)~log(DescentRate),

data=dist.narm)

dist.9=lm(log(DistFrmTower)~log(Wingloading)+log(DescentRate),

data=dist.narm)

dist.10=lm(log(DistFrmTower)~log(Area)+log(DescentRate),

data=dist.narm)

dist.11=lm(log(DistFrmTower)~log(Mass)+log(DescentRate),

data=dist.narm)

dist.12=lm(log(DistFrmTower)~log(Mass)+log(Area)+log(DescentRate),

data=dist.narm)

# Compare the AIC values for these models:

distanceAIC=AIC(dist.1,dist.2,dist.3,dist.4,dist.5,dist.6,

dist.7,dist.8,dist.9,dist.10,dist.11,dist.12)

# Order by AIC value (smallest to largest)

distanceAIC=distanceAIC[order(distanceAIC$AIC),]

distanceAIC$dAIC <- distanceAIC$AIC-distanceAIC$AIC[1]

# dist.9, dist.12, and dist.11 are the best models. View them and calculate R-squared values:

summary(dist.9)

summary(dist.12)

summary(dist.11)

summary(dist.3)

summary(dist.6)

summary(dist.8)

summary(dist.10)

summary(dist.5)

summary(dist.2)

Fixed.Rsquared (log(fruits.narm$DistFrmTower[!is.na(fruits.narm$DistFrmTower)]),

fitted(dist.2),1)

summary(dist.1)

summary(dist.4)

summary(dist.7)

Fixed.Rsquared (log(fruits.narm$DistFrmTower[!is.na(fruits.narm$DistFrmTower)]),

fitted(dist.7),1)

## 5. Compare models to predict mean, median, and qualtiles of rate of dispersal distance for

## each tree.

## Models for the mean of descent rate

# dist.mean1: First principles: square-root of wingloading.

# dist.mean2: First principles: inverse of descent rate.

# dist.mean3: The best model predicted from individual seed data

dist.mean1=dist.2$coefficients[1]-0.5*wl.summary$mean

dist.mean2=dist.7$coefficients[1]-dr.summary$mean

dist.mean3=dist.9$coefficients[1]+dist.9$coefficients[2]*wl.summary$mean+dist.9$coefficients[3]*dr.summary$mean

# Calculate Pearson correlation coefficient and significance

cortest1 <- cor.test(dist.summary$mean, dist.mean1,method='pearson',exact=T)

cortest1$estimate; cortest1$p.value

cortest2 <- cor.test(dist.summary$mean, dist.mean2,method='pearson',exact=T)

cortest2$estimate; cortest2$p.value

cortest3 <- cor.test(dist.summary$mean, dist.mean3,method='pearson',exact=T)

cortest3$estimate; cortest3$p.value

## Models for the median of descent rate

# dist.q501: First principles: square-root of wingloading.

# dist.q502: First principles: inverse of descent rate.

# dist.q503: The best model predicted from individual seed data

dist.q501=dist.2$coefficients[1]-0.5*wl.summary$q50

dist.q502=dist.7$coefficients[1]-dr.summary$q50

dist.q503=dist.9$coefficients[1]+dist.9$coefficients[2]*wl.summary$q50+dist.9$coefficients[3]*dr.summary$q50

# Calculate Pearson correlation coefficient and significance

cortest1 <- cor.test(dist.summary$q50, dist.q501,method='pearson',exact=T)

cortest1$estimate; cortest1$p.value

cortest2 <- cor.test(dist.summary$q50, dist.q502,method='pearson',exact=T)

cortest2$estimate; cortest2$p.value

cortest3 <- cor.test(dist.summary$q50, dist.q503,method='pearson',exact=T)

cortest3$estimate; cortest3$p.value

## Models for the 75th quartile of descent rate

# dist.q751: First principles: square-root of wingloading.

# dist.q752: First principles: inverse of descent rate.

# dist.q753: The best model predicted from individual seed data

dist.q751=dist.2$coefficients[1]-0.5*wl.summary$q75

dist.q752=dist.7$coefficients[1]-dr.summary$q75

dist.q753=dist.9$coefficients[1]+dist.9$coefficients[2]*wl.summary$q75+dist.9$coefficients[3]*dr.summary$q75

# Calculate Pearson correlation coefficient and significance

cortest1 <- cor.test(dist.summary$q75, dist.q751,method='pearson',exact=T)

cortest1$estimate; cortest1$p.value

cortest2 <- cor.test(dist.summary$q75, dist.q752,method='pearson',exact=T)

cortest2$estimate; cortest2$p.value

cortest3 <- cor.test(dist.summary$q75, dist.q753,method='pearson',exact=T)

cortest3$estimate; cortest3$p.value

## Models for the 90th quartile of descent rate

# dist.q901: First principles: square-root of wingloading.

# dist.q902: First principles: inverse of descent rate.

# dist.q903: The best model predicted from individual seed data

dist.q901=dist.2$coefficients[1]-0.5*wl.summary$q90

dist.q902=dist.7$coefficients[1]-dr.summary$q90

dist.q903=dist.9$coefficients[1]+dist.9$coefficients[2]*wl.summary$q90+dist.9$coefficients[3]*dr.summary$q90

# Calculate Pearson correlation coefficient and significance

cortest1 <- cor.test(dist.summary$q90, dist.q901,method='pearson',exact=T)

cortest1$estimate; cortest1$p.value

cortest2 <- cor.test(dist.summary$q90, dist.q902,method='pearson',exact=T)

cortest2$estimate; cortest2$p.value

cortest3 <- cor.test(dist.summary$q90, dist.q903,method='pearson',exact=T)

cortest3$estimate; cortest3$p.value

## Models for the 95th quartile of descent rate

# dist.q951: First principles: square-root of wingloading.

# dist.q952: First principles: inverse of descent rate.

# dist.q953: The best model predicted from individual seed data

dist.q951=dist.2$coefficients[1]-0.5*wl.summary$q95

dist.q952=dist.7$coefficients[1]-dr.summary$q95

dist.q953=dist.9$coefficients[1]+dist.9$coefficients[2]*wl.summary$q95+dist.9$coefficients[3]*dr.summary$q95

# Calculate Pearson correlation coefficient and significance

cortest1 <- cor.test(dist.summary$q95, dist.q951,method='pearson',exact=T)

cortest1$estimate; cortest1$p.value

cortest2 <- cor.test(dist.summary$q95, dist.q952,method='pearson',exact=T)

cortest2$estimate; cortest2$p.value

cortest3 <- cor.test(dist.summary$q95, dist.q953,method='pearson',exact=T)

cortest3$estimate; cortest3$p.value

## Models for the maximum descent rate

# dist.q1001: First principles: square-root of wingloading.

# dist.q1002: First principles: inverse of descent rate.

# dist.q1003: The best model predicted from individual seed data

dist.q1001=dist.2$coefficients[1]-0.5*wl.summary$q100

dist.q1002=dist.7$coefficients[1]-dr.summary$q100

dist.q1003=dist.9$coefficients[1]+dist.9$coefficients[2]*wl.summary$q100+dist.9$coefficients[3]*dr.summary$q100

# Calculate Pearson correlation coefficient and significance

cortest1 <- cor.test(dist.summary$q100, dist.q1001,method='pearson',exact=T)

cortest1$estimate; cortest1$p.value

cortest2 <- cor.test(dist.summary$q100, dist.q1002,method='pearson',exact=T)

cortest2$estimate; cortest2$p.value

cortest3 <- cor.test(dist.summary$q100, dist.q1003,method='pearson',exact=T)

cortest3$estimate; cortest3$p.value

**R script 3 for Mechanistic models**

###### Intraspecific variation in seed dispersal of a Neotropical tree and its relationship to

###### fruit and tree traits.

###### Augspurger et al.

###### Mechanistic models.R ######

#### This file fits mechanistic models to predict dispersal distances from fruit and tree traits, and

#### calculates correlations between distances predicted from the mechanistic model and measured in

#### the field.

# Load required packages

library(alphahull)

library(splancs)

# Load data files for parent trees, individual fruits, and seed shadows.

tree.data <- read.csv('Parent tree information.csv')

fruit.data <- read.csv('Individual fruits.csv')

shadow.data <- read.csv('Seed shadows.csv')

# Load data file with grid of positions within the measured crown area of each tree.

load('~/Documents/STRI/Platy paper/anisotropic plots/tree.grid.RData')

# Load wind data. In this data file, each day of data is a separate item in the list called

# 'WindDataList'. Ux and Uy are the horizontal wind velocities in the x- and y- directions,

# and Uz is the vertical wind velocity.

load("~/Documents/STRI/Platy paper/Wind data/WindData.RData")

for(i in 1:length(WindDataList)) {

WindDataList[[i]]$Ux <- as.numeric(WindDataList[[i]]$Ux)

WindDataList[[i]]$Uy <- as.numeric(WindDataList[[i]]$Uy)

WindDataList[[i]]$Uz <- as.numeric(WindDataList[[i]]$Uz)

}

# Define the WINDISPER-E model, as formulated in:

# Nathan, R., Horn, H. S., Chave, J., and S.A. Levin. 2002. Mechanistic models for tree seed

# dispersal by wind in dense forests and open landscapes. Pg. 69-82 In: D.J. Levey, W.R. Silva

# and M. Galetti (eds.) Seed Dispersal and Frugivory: Ecology, Evolution and Conservation.

# CAB International, Wallingford, UK.

# Model parameters:

# UHc = horizontal windspeed at the height of the canopy (m/s)

# Hc = height of the canopy (m)

# alpha = attenuation coefficient (unitless)

# Tv = terminal velocity

# Uv = vertical windspeed (m/s)

# H = height of seed release (m)

dist.function <- function(UHc,Hc,alpha,Tv,Uv,H){

coeff <- (UHc*Hc)/(alpha*(Tv-Uv))

e.val <- alpha*(H-Hc)/Hc

D.value <- coeff*(exp(e.val)-exp(-alpha))

return(D.value)

}

# For each tree, simulate 50,000 seeds

nseeds <- 50000

modelD.results <- list() #Create a list to store results from each of 6 parent trees

for (i in 1:6){

fruits.i <- fruit.data[fruit.data$Year==1983 & fruit.data$Parent==tree.data$Parent[i],]

# Canopy height: assume constant value for all trees

Hc.i <- 22

# Attenuation coefficient: assume constant for all trees

alpha.i <- 3

# Terminal velocity: sample from descent rates

Tv.i <- sample(fruits.i[!is.na(fruits.i$DescentRate),'DescentRate'],size=nseeds,

replace=T)

# Height of seed release: sample from distance between bottom and top of canopy

H.i <- sample(seq(from=tree.data$MinCrownHt[i], to=tree.data$MaxCrownHt[i],length=1000),

size=nseeds,replace=T)

# Vertical wind speed: assume equal to zero

Uv.i <- 0

# Sample wind data to find: horizontal wind speed and direction for each of j seeds

UHc.i <- c()

Dir.i <- c()

for (j in 1:nseeds){

# calculate time to average based on tree height and descent rate of seed

timej <- ceiling(H.i[j]/Tv.i[j])

# sample a day of wind data

wind.data <- WindDataList[[sample(1:length(WindDataList),size=1)]]

# choose starting index (exclude entries at the end that won't have enough time after)

samplej <- sample(c(1:(length(wind.data$TIMESTAMP)-timej*10)),size=1)

# x-values

Uxj <- wind.data[samplej:(samplej+timej*10),'Ux']

# y-values

Uyj <- wind.data[samplej:(samplej+timej*10),'Uy']

# z-values

Uzj <- wind.data[samplej:(samplej+timej*10),'Uz']

# calculate average horizontal speed

UHc.i[j] <- sqrt(mean(Uxj, na.rm=T)^2+mean(Uyj, na.rm=T)^2)

# calculate wind direction

theta <- atan2(mean(Uyj, na.rm=T),mean(Uxj, na.rm=T))

Dir.i[j] <- theta/pi*180

}

# Make sure direction is between 0 and 360 degrees

Dir.i <- ifelse(Dir.i < 360,

Dir.i + 360,

Dir.i)

Dir.i <- ifelse(Dir.i > 360,

Dir.i - 360,

Dir.i)

# calculate dispersal distance using mechanistic model

D.i <- dist.function(UHc.i,Hc.i,alpha.i,Tv.i,Uv.i,H.i)

# Sample starting position from within tree canopy, then use distance and direction to calculate

# ending position

position.index <- sample(c(1:length(tree.grid[[i]]$x)),size=nseeds,replace=T)

start.x <- tree.grid[[i]]$x[position.index]

start.y <- tree.grid[[i]]$y[position.index]

seed.x <- start.x - D.i*cos(Dir.i*pi/180)

seed.y <- start.y - D.i*sin(Dir.i*pi/180)

mech.results <- data.frame(Dir.i, D.i, seed.x, seed.y)

modelD.results[[i]] <- mech.results

}

# Calculate Pearson correlation coefficient and significance between modeled distances and

# observed distances.

# Load summary results from one simulation of 50,000 seeds per tree.

mech.results <- read.csv('Mechanistic model results.csv')

# Compare modeled distance from the trunk and observed distance from the trunk:

# mean

cortest.mean <- cor.test(mech.results$Trunk.mean, shadow.data$Obs.dist.mean,

method='pearson',exact=T)

cortest.mean$estimate; cortest.mean$p.value

# median

cortest.p50 <- cor.test(mech.results$Trunk.p50, shadow.data$Obs.dist.50,

method='pearson',exact=T)

cortest.p50$estimate; cortest.p50$p.value

# 75th percentile

cortest.p75 <- cor.test(mech.results$Trunk.p75, shadow.data$Obs.dist.75,

method='pearson',exact=T)

cortest.p75$estimate; cortest.p75$p.value

# 90th percentile

cortest.p90 <- cor.test(mech.results$Trunk.p90, shadow.data$Obs.dist.90,

method='pearson',exact=T)

cortest.p90$estimate; cortest.p90$p.value

# 95th percentile

cortest.p95 <- cor.test(mech.results$Trunk.p95, shadow.data$Obs.dist.95,

method='pearson',exact=T)

cortest.p95$estimate; cortest.p95$p.value

# Maximum

cortest.p100 <- cor.test(mech.results$Trunk.p100, shadow.data$Obs.dist.max,

method='pearson',exact=T)

cortest.p100$estimate; cortest.p100$p.value

# total area

cortest.area <- cor.test(mech.results$Area, shadow.data$Area,

method='pearson',exact=T)

cortest.area$estimate; cortest.area$p.value

# Compare modeled distance from a point source and fitter kernel distance from a point source:

# mean

cortest.mean <- cor.test(mech.results$Pt.mean, shadow.data$Mod.dist.mean,

method='pearson',exact=T)

cortest.mean$estimate; cortest.mean$p.value

# median

cortest.p50 <- cor.test(mech.results$Pt.p50, shadow.data$Mod.dist.50,

method='pearson',exact=T)

cortest.p50$estimate; cortest.p50$p.value

# 75th percentile

cortest.p75 <- cor.test(mech.results$Pt.p75, shadow.data$Mod.dist.75,

method='pearson',exact=T)

cortest.p75$estimate; cortest.p75$p.value

# 90th percentile

cortest.p90 <- cor.test(mech.results$Pt.p90, shadow.data$Mod.dist.90,

method='pearson',exact=T)

cortest.p90$estimate; cortest.p90$p.value

# 95th percentile

cortest.p95 <- cor.test(mech.results$Pt.p95, shadow.data$Mod.dist.95,

method='pearson',exact=T)

cortest.p95$estimate; cortest.p95$p.value

# Maximum

cortest.p100 <- cor.test(mech.results$Pt.p100, shadow.data$Mod.dist.max,

method='pearson',exact=T)

cortest.p100$estimate; cortest.p100$p.value
